# Pheno-seq – linking visual features and gene expression in 3D cell culture systems

**DOI:** 10.1038/s41598-019-48771-4

**Published:** 2019-08-26

**Authors:** Stephan M. Tirier, Jeongbin Park, Friedrich Preußer, Lisa Amrhein, Zuguang Gu, Simon Steiger, Jan-Philipp Mallm, Teresa Krieger, Marcel Waschow, Björn Eismann, Marta Gut, Ivo G. Gut, Karsten Rippe, Matthias Schlesner, Fabian Theis, Christiane Fuchs, Claudia R. Ball, Hanno Glimm, Roland Eils, Christian Conrad

**Affiliations:** 1 Digital Health Center, Berlin Institute of Health (BIH)/Charité-Universitätsmedizin Berlin, Berlin, Germany; 2 Center for Quantitative Analysis of Molecular and Cellular Biosystems (BioQuant), University of Heidelberg, Heidelberg, Germany; 3 Division of Theoretical Bioinformatics, German Cancer Research Center (DKFZ), Heidelberg, Germany; 4 Present Address: Max Delbrück Center for Molecular Medicine, Berlin Institute for Medical Systems Biology, Berlin, Germany; 5 Helmholtz Zentrum München - German Research Center for Environmental Health, Institute of Computational Biology, Munich, Neuherberg Germany; 6 Department of Mathematics, Technische Universität München, Munich, Germany; 7 Present Address: Division of Chromatin Networks, German Cancer Research Center (DKFZ), Heidelberg, Germany; 8 Heidelberg Center for Personalized Oncology, DKFZ-HIPO, DKFZ, Heidelberg, Germany; 9 CNAG-CRG, Centre for Genomic Regulation, Barcelona Institute of Science and Technology, Barcelona, Spain; 10 Universitat Pompeu Fabra, Barcelona, Spain; 11 Bioinformatics and Omics Data Analytics, German Cancer Research Center (DKFZ), Heidelberg, Germany; 12 Faculty of Business Administration and Economics, Bielefeld University, Bielefeld, Germany; 13 Department of Translational Oncology, NCT Dresden, University Hospital, Carl Gustav Carus, Technische Universität Dresden, Dresden and DKFZ, Heidelberg, Germany; 14 German Cancer Consortium, Heidelberg, Germany; 15 Health Data Science Unit, University Hospital Heidelberg, Heidelberg, Germany

**Keywords:** Fluorescence imaging, Transcriptomics, Cancer models

## Abstract

Patient-derived 3D cell culture systems are currently advancing cancer research since they potentiate the molecular analysis of tissue-like properties and drug response under well-defined conditions. However, our understanding of the relationship between the heterogeneity of morphological phenotypes and the underlying transcriptome is still limited. To address this issue, we here introduce “pheno-seq” to directly link visual features of 3D cell culture systems with profiling their transcriptome. As prototypic applications breast and colorectal cancer (CRC) spheroids were analyzed by pheno-seq. We identified characteristic gene expression signatures of epithelial-to-mesenchymal transition that are associated with invasive growth behavior of clonal breast cancer spheroids. Furthermore, we linked long-term proliferative capacity in a patient-derived model of CRC to a lowly abundant PROX1-positive cancer stem cell subtype. We anticipate that the ability to integrate transcriptome analysis and morphological patho-phenotypes of cancer cells will provide novel insight on the molecular origins of intratumor heterogeneity.

## Introduction

Three-dimensional (3D) cell culture systems (e.g. spheroids^1^, organoids^2^) are characterized by self-organizing multicellular structures that reflect critical physiologic features of tissue geometry, cellular interactions and disease^3^. Therefore, they provide a relevant context for *in-vitro* testing of single cell behavior. During maturation in 3D culture, single cells undergo several rounds of replication accompanied by morphological and functional changes that rely on underlying gene expression programs. Depending on the initial single cell state, the resulting visual spheroid/organoid phenotype(s) can be highly informative for heterogeneous cellular functions^4–6^ as well as for classification of tumor subtypes and disease states^7,8^.

In particular, individual cancer cells obtained from the same tumor sample and grown under the same conditions frequently exhibit strong differences in replicative potential^4^, invasive behavior^9^ and drug responses^10^. This may be attributed to genetic diversity and clonal evolution^11^, epigenetic alterations^12^, microenvironmental influences^13^ or stochastic gene expression^14^. This phenomenon of ‘intratumor heterogeneity’ is emerging as an essential driver of tumorigenic progression, treatment resistance and relapse^15^.

A deeper understanding of morphological heterogeneity between clonal spheroids or organoids derived from a single patient requires the parallel acquisition of system-wide gene expression information. On the one hand, technologies for single cell RNA-seq (scRNA-seq)^16,17^ have greatly improved the analysis of intratumor heterogeneity by enabling the unbiased detection of transcript abundances in individual cells^18–20^. Notably, these approaches do not provide a direct link to visual cellular phenotypes since the available protocols involve dissociation of cells and loss of their multicellular context. On the other hand, several powerful methods combining imaging and sequencing have been developed lately that enable transcriptomic profiling *in-situ* at high cellular resolution^21–25^. However, these methods require histological preparation which complicates or even prevents combined image-based and transcriptional profiling of one intact clonal spheroid or organoid. In addition, state-of-the-art methods for spatial transcriptomics require highly complex experimental setups^23–25^ which limits broader applicability.

A recent landmark study highlighted the importance of directly combining imaging and sequencing in 3D cell culture systems by dissecting morphological and functional heterogeneities from clonal intestinal organoids^6^, but yet without directly matching image and transcriptional features from the same organoid.

To address the abovementioned issues, we here introduce ‘pheno-seq’ to dissect cellular heterogeneity in 3D cell culture systems by directly combining clonal cell culture, imaging and transcriptomic profiling without histological preparation. Pheno-seq represents a new transcriptome analysis strategy that complements existing bulk and scRNA-seq approaches and enables a direct match of image features and gene expression in single clonal spheroids. We developed an experimental and computational workflow for high-throughput pheno-seq, including automated dispensing and imaging of single spheroids in barcoded nanowells as well as an automated image processing pipeline. We demonstrate the utility of pheno-seq in dissecting both morphological and transcriptional heterogeneity for established and patient-derived 3D-models of breast and colon cancer, respectively.

## Results

### Pheno-seq directly links visual phenotypes and gene expression in 3D cell culture systems

We established the pheno-seq method using the MCF10CA cell line, a transformed derivative of the MCF10 progression line^26^. MCF10 cell lines reflect morphological phenotypes of epithelial breast cancer, in which normal epithelial cells undergo a stepwise transformation from local hyperplasia to premalignant carcinoma *in-situ* and invasive carcinoma^27^. The non-neoplastic parental cell line MCF10A forms polarized acinar spheroids closely resembling the lobular structures of the mammary gland^28^. In contrast, MCF10CA^29^ cells have invasive and metastatic properties in xenografts^30^. Similarly, clonal MCF10CA spheroids display heterogeneous morphologies reflecting characteristics of late stages of breast cancer carcinomas, including ‘round’ (*in-situ*) and ‘aberrant’ (invasive) phenotypes (Supplementary Fig. 1a,b). With this cellular system, the pheno-seq protocol was established that consists of the following steps: (i) *Isolation and functional analysis of spheroid phenotypes*, (ii) *Data acquisition*, and (iii) *Integrative analysis of morphological phenotypes and transcriptome*.

*Isolation and functional analysis of spheroid phenotypes*. We developed a protocol to isolate single spheroids from reconstituted basement membrane (Matrigel) without perturbing their phenotypic identity (see Methods). We functionally analyzed the observed morphological heterogeneity by reseeding and culturing cells from both phenotype classes independently (‘round’ and ‘aberrant’). Quantitative image analysis revealed enriched morphology appearances for each reseeded phenotype class indicating for high cell state stability and efficient isolation of different phenotype classes (Supplementary Fig. 1c).

**Figure 1:**
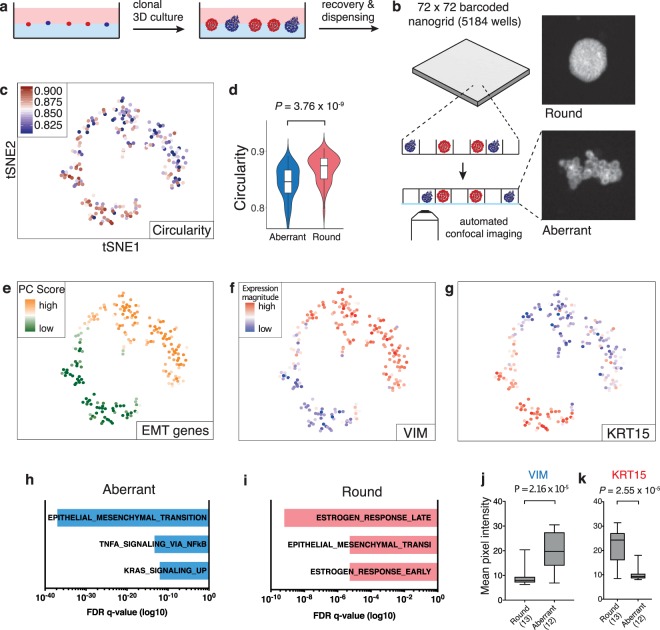
Pheno-seq directly links visual phenotypes and gene expression in 3D culture systems at high-throughput. (**a**) Workflow overview for the culture and recovery of clonal spheroids for inference of morphology-specific gene expression. (**b**) Pheno-seq workflow based on automated dispensing and confocal imaging of recovered spheroids stained by CellTrackerRed in barcoded nanowells. (**c**) 2D tSNE visualization of 210 pheno-seq 3′-end RNA-seq profiles with coloring based on image feature ‘circularity’. For better visualization, all circularity values below 0.8 were set to minimum in the color code scheme. (**d**) Spheroid circularity plotted per cluster (k-means clustering, k = 2) as shown in (**c**). Violin-plot center-line: median; box limits: first and third quartile; whiskers: ±1.5 IQR. Indicated *P*-value from unpaired two-tailed Students t-test. (**e**) Same 2D tSNE visualization as shown in (**c**) with coloring based on PAGODA’s PC scores for HALLMARK_EMT gene set derived from the Molecular Signature Database (MSigDB)^38^. (**f**,**g**) Same 2D tSNE visualization as shown in (**c**) with coloring based on expression magnitude for EMT marker VIM (**f**) and epithelial marker KRT15 (**g**). (**h**,**i**) Gene set enrichment analysis based on Hallmark gene sets^35^ for ‘aberrant’ (**h**) and ‘round’ (**i**) phenotype specific genes identified by differential expression analysis^37^ derived from the MSigDB. Bar plots show top three enriched gene sets ranked by FDR q-values. Example genes are VIM, TGFA, FAP for ‘aberrant’ and KRT15, CA2 and KRT16 for ‘round’ phenotypes. (**j**,**k**) Validation of phenotype-specific expression for VIM (aberrant) and KRT15 (round) by whole mount immunofluorescence (IF). Plotted values reflect mean pixel intensity per classified spheroid. Box plot center-line: median; box limits: first and third quartile; whiskers: min/max values. Numbers of samples indicated on x-axis under respective phenotype class. Indicated are *P*-values from unpaired two-tailed Students t-test.

*Data acquisition*. Pheno-seq combines automated imaging and transcriptomic profiling of individual clonal spheroids in a single workflow. This was achieved by repurposing the nanowell-based iCELL8 single cell sequencing system^31^ for the processing of spheroid samples of up to 150 µm in size. Key modifications for accurate spheroid image profiling and subsequent processing for RNA-seq included cellular fixation^32^, altered chip setup, higher-resolution microscopy, an automated image-processing pipeline and an interactive webtool for analysis and selection of spheroids for sequencing (Supplementary Figs 2 and 3, Fig. 1a,b). RNA-seq was conducted with the standard iCELL8 protocol that includes reverse transcription and cDNA amplification in nanowells as well as pooled 3′-end sequencing library preparation. With this setup, MCF10CA pheno-seq profiles of 210 spheroids were acquired.

*Integrative analysis of morphological phenotypes and transcriptome*. We analyzed MCF10CA pheno-seq profiles (n = 210 spheroids) by testing annotated and *de-novo* identified gene sets for coordinated expression variability^33^. 2D t-SNE visualization of RNA-seq data revealed two distinct clusters of spheroids that also differed strongly in several morphological image features (Fig. 1c,d and Supplementary Fig. 4). In particular, the observed heterogeneity in ‘circularity’ (Fig. 1c,d) is of primary interest as it informs about the epithelial integrity of epithelial/round spheroid (high circularity values) and more transformed invasive/aberrant phenotypes (low circularity values). Thus, these results indicate that the major transcriptional heterogeneity between spheroids relates to observed differences in morphological characteristics.

### In depth analysis of MCF10CA pheno-seq data

Pheno-seq provides a wealth of data. In dependence of the specific cellular systems different biological questions can be addressed. For the MCF10CA system, we further investigated the molecular changes accompanying the transition from epithelial to invasive behavior. In breast cancer cells, this generally involves a specific gene expression program described as ‘epithelial-to-mesenchymal transition’ (EMT)^34^. Similarly, the spheroid cluster with low circularity values (‘aberrant’ phenotype) is defined by expression of an EMT signature^35^, including the major mesenchymal marker vimentin (VIM). In contrast, the cluster with highly circular spheroids (‘round’ phenotype) is characterized by high expression of KRT15, a basal-myoepithelial marker in the mammary gland^36^ (Fig. 1e–g). Differential expression analysis^37^ (fold change >1.3; adjusted p-value < 0.1) and gene set enrichment analysis^35,38^ revealed concordant results. EMT related genes were highly enriched in aberrant spheroids, including VIM, JUN, RHOB, VCAN and FAP, respectively. Conversely, round phenotypes exhibit high expression of epithelial markers (e.g. KRT15, KRT16, KRT23 and DSG3) as well as genes involved in the response to the primary female sex hormone Estrogen (e.g. CA2, FABP5 and AGR2) (Fig. 1h,i). We validated spheroid phenotype-specific expression for VIM and KRT15 by quantitative immunofluorescence (Fig. 1j,k and Supplementary Fig. 5).

### Comparison of pheno-seq and scRNA-seq data

Full-length pheno-seq (n = 8) and scRNA-seq (n = 166, reflecting approx. 6 spheroids) based on manually isolated spheroids yielded similar results with two distinct clusters that show a tight association of spheroids to their original phenotype class (Supplementary Fig. 6a–f). Notably, only pheno-seq enables a direct and quantitative association of image and transcriptomic features as standard scRNA-seq protocols requires multiple spheroids (>40 of each class) to achieve sufficiently high input material for cell capture.

Pheno-seq resulted in higher gene detection rates per sample compared to scRNA-seq, most likely due to the enhanced RNA-input that comprised a higher number of cells (Supplementary Fig. 6g, Supplementary Table 1). Surprisingly, we could not detect the major round-specific marker KRT15 by differential expression analysis of scRNA-seq data, even by generating synthetic pheno-seq profiles from averaged single-cell expression (Supplementary Fig. 7). This phenomenon could be due to the lower gene detection rate of scRNA-seq (Supplementary Fig. 6g) or due to the prolonged dissociation and processing procedure that can induce biases in detecting marker genes^39^.

### Application of pheno-seq to a patient-derived colorectal cancer model

We next set out to assess the functional association of visual phenotypes and gene expression in a clinically relevant and more complex 3D model of colorectal cancer (CRC)^4^. Dieter *et al*. identified and characterized functionally distinct subtypes of patient-derived CRC cells both *in vitro* and *in vivo* that differed in their long-term proliferative capacity^4^. The reported heterogeneity seems to be largely independent of mutational subclone diversity^40^, indicating the presence of a differentiation-like hierarchy in CRC^41^. However, which lineage-related subtypes actually confer proliferative capacity has not been investigated, yet.

Single CRC cells form different spheroid phenotypes in terms of their replicative potential (Supplementary Fig. 8a). These include long-term proliferating, transient amplifying and postmitotic subtypes. In order to test whether spheroid forming capacity of single cells is associated with the proliferative capacity of their spheroid of origin, we reseeded cells derived from clonal spheroids that strongly differed in size after 10 days (20–40 µm vs. 70–100 µm). Quantitative image analysis revealed significant differences in spheroid forming capacity (Supplementary Fig. 8b), indicating that different sizes of clonal spheroids are associated with different compositions of functionally distinct subtypes. Moreover, reseeding of big spheroids resulted in mixed phenotypes (Supplementary Fig. 8a), in line with the hierarchical cancer stem cell model of CRC.

**Figure 2:**
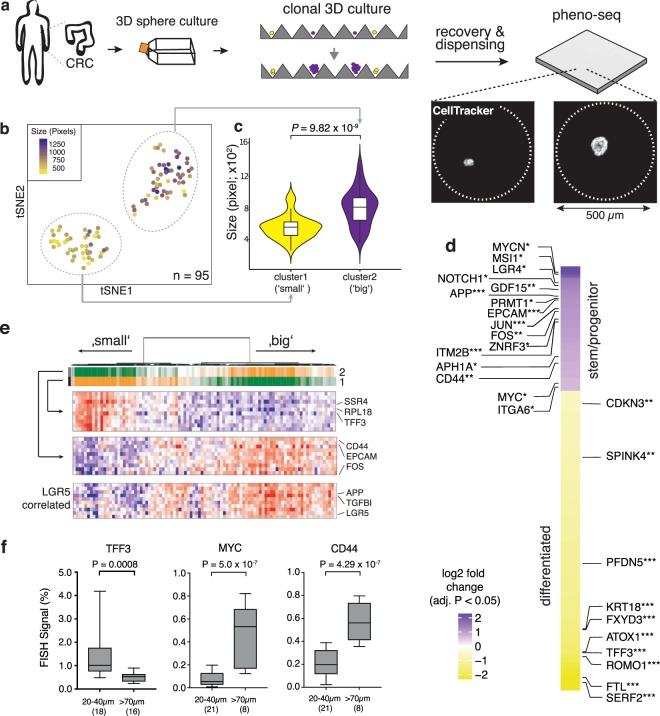
Pheno-seq with a 3D model of colorectal cancer links heterogeneous proliferative phenotypes to expression signatures enriched for lineage-specific markers. (**a**) Clonal 3D-culture in inverse pyramidal shaped microwells and recovery strategy for HT-pheno-seq of patient-derived CRC spheroids isolated from a liver metastasis. Yellow and purple indicate heterogeneous subpopulations with functional differences in proliferative capacity^4^. (**b**) 2D tSNE visualization of 95 HT-pheno-seq expression profiles. Coloring by sphere size (pixel). (**c**) Spheroid size plotted per cluster. Violin-plot center-line: median; box limits: first and third quartile; whiskers: ±1.5 IQR). Indicated *P*-value calculated from unpaired two-tailed Students t-test. (**d**) Heatmap reflecting differential expression analysis^37^ of identified clusters in (**b**). Selected genes are listed beside the heatmap; Fold change >1.5; adjusted *P*-value < 0.05; *P < 0.05, **P < 0.01, ***P < 0.001; ‘small’ cluster1: 313 differentially expressed genes; ‘big’ cluster: 130 differentially expressed genes. (**e**) PAGODA RNA-seq analysis heatmap of CRC spheroid pheno-seq data. Dendrogram reflects overall clustering and the rows below represent top two significant aspects of heterogeneity based on HALLMARK/GO gene sets derived from the MSigDB^38^ and on *de-novo* identified gene sets. High PC Scores correspond to high expression of associated gene sets. Expression patterns below reflect top 10 loading genes for selected gene sets that are associated with respective aspects. Bottom: Expression pattern of genes most highly correlated with intestinal stem cell marker LGR5 (Pearson’s correlation). (**f**) Validation of pheno-seq by quantitative RNA-FISH for size-dependent differentiation marker TFF3 and cancer stem cell markers CD44/MYC. Plotted values reflect the pixel fraction that exceeds the background threshold per spheroid (Box plot center-line: median; box limits: first and third quartile; whiskers: min/max values; *P*-values from unpaired Students t-test. Numbers of samples n indicated on x-axis under respective class).

To identify transcriptional signatures that distinguish these heterogenous proliferative phenotypes, we performed pheno-seq based on clonal CRC spheroids cultured in a microwell setup (Fig. 2a, Supplementary Fig. 8c). Analysis of 95 HT-pheno-seq RNA-seq profiles and t-SNE visualization^33^ confirmed two transcriptionally distinct clusters (Fig. 2b). Associated image analysis revealed a strong difference in spheroid size between both clusters (Fig. 2b,c).

### Assignment of lineage-related genes to heterogeneous CRC growth phenotypes

We reasoned that the two different transcriptome types define spheroids that originated either from long-term proliferating (‘big’ phenotype) or transit-amplifying cells (‘small phenotype’). Differential expression analysis^37^ showed that the first cluster (‘small’-phenotype) is enriched for secretory intestinal differentiation markers, including TFF3, KRT18 and SPINK4^42^ (Fig. 2d). In contrast, the second cluster (‘big’-phenotype) is characterized by the expression of genes previously described to be involved in intestinal stem/progenitor cell maintenance (e.g., CD44, MYC, NOTCH1, APP, MSI1 and ITGA6)^42,43^, the formation of cell-cell junctions (e.g., EPCAM, CLDN4, CDH1) and WNT signaling (ZNRF3, LGR4, JUN). In addition, we identified several genes related to the γ-secretase machinery (e.g., NOTCH1, APP, ITM2B, APH1A and CD44) a key component of the Notch signaling pathway and target of novel therapies that aim to disrupt cancer stem cell signaling^44^ (Fig. 2d). Finally, the pattern of this cluster-specific signature showed a high overlap with genes correlated with the major intestinal stem cell marker LGR5^41^ (Fig. 2e). We could validate sphere size-dependent expression for selected lineage-specific markers by quantitative RNA-FISH (Fig. 2f, Supplementary Fig. 9).

Thus, pheno-seq is able to directly assign lineage-related genes to heterogeneous growth phenotypes. Furthermore, our results support the hypothesis of a hierarchical organization in CRC with a stem/progenitor-like cell population at the apex.

### Single-cell deconvolution of CRC pheno-seq data

In order to increase the resolution of the pheno-seq transcriptome data and to computationally infer single-cell regulatory states we combined image analysis and gene expression deconvolution. First, cell numbers from CRC pheno-seq imaging were determined from the relationship of spheroid size and nuclei counts using light-sheet microscopy and 3D image analysis (Supplementary Fig. 10a). As the original pheno-seq data exhibited a poor association between library complexity and estimated cell numbers (Supplementary Fig. 10b), we downsampled the data to achieve a constant number of mRNA counts per estimated single cell content (Supplementary Fig. 10c). As expected, this approach introduced a positive correlation of cell numbers to housekeeping genes (e.g. ACTB) with a constant number of mRNA molecules per cell (Supplementary Fig. 10d). However, the heterogeneously expressed differentiation marker TFF3 does not exhibit any correlation with cell numbers, demonstrating the suitability of our normalization approach.

**Figure 3:**
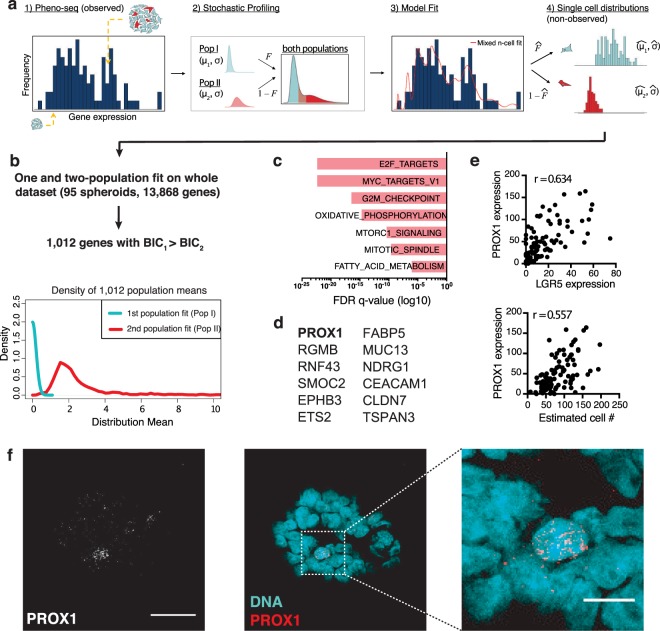
Single-cell deconvolution of CRC spheroid pheno-seq data by maximum likelihood inference. (**a**) Concept of adapted maximum likelihood approach^45^ based on estimated cell numbers and transformed pheno-seq data (n = 95): (1) Acquired and transformed pheno-seq data based on estimated cell numbers build a distribution of measurements for inference by the model. Coloring of cells in spheroids: red = stem-like; cyan = differentiated. (2) Assumptions on single cell distributions: Model of heterogeneous gene regulation in which single cells are supposed to exhibit gene expression at low (Pop I) or high (Pop II) levels with a common coefficient of variation. The four parameters of the model are the log-mean expression for each subpopulation (𝜇_1_ and 𝜇_2_), the proportion of cells in the high subpopulation (𝐹), and the common log-SD of expression (σ). (3) Based on the model in step 2, a likelihood function is derived that takes different numbers of cells per spheroid into account. The likelihood function is then maximized by searching through the four parameters of the model to identify those that are most likely given the experimental observations. 4) These four parameters define the inferred single cell distributions of the low and high-level populations. (**b**) 1,012 genes show an improved two-population fit compared to a one population fit (BIC: Bayesian information criterion). Densities of the means of the first (Pop I: low regulatory state) and second population (Pop II: high regulatory state) for all identified 1,012 genes. (**c**) Gene set enrichment analysis for two-population genes based on Hallmark gene sets^35^ derived from the MSigDB^38^. Bar plot showing top enriched gene sets ranked by FDR q-values. (**d**) Selected human colonic stem and differentiation markers^46^ that have been identified by pheno-seq deconvolution. (**e**) Scatter plots for relations of PROX1 expression and estimated cell numbers (lower) and between PROX1 expression and expression of the major intestinal stem cell marker LGR5 (upper) as well as associated Pearson’s correlation coefficients (r). (**f**) RNA-FISH staining of CRC spheroids for PROX1 (Atto550) and DAPI counterstaining for visualization of DNA. Merged images: DNA: cyan; PROX1: red. Images represent Z-projections (scale bar 30 µm and 10 µm for magnified merged image).

Next, we identified genes whose expression originates from heterogeneous single-cell regulatory states. A maximum likelihood inference approach initially developed to deconvolve cell-to-cell heterogeneities from random 10-cell samples^45^ was used (Fig. 3a). Deconvolution of the entire CRC pheno-seq dataset revealed 1,012 genes that show an improved two-population fit compared to a one-population fit, assessed by the Bayesian information criterion (BIC) to calculate the quality of the fit relative to the number of inferred parameters (Fig. 3b). Gene set enrichment analysis revealed a high proportion of MYC targets as well as genes involved in the regulation of cell growth and proliferation (Fig. 3c). Strikingly, several identified genes are overlapping with murine and human intestinal stem cell markers revealed by scRNA-seq^42,46^, including SMOC2, RGMB, APP, MAPK1, EPHB3 and RNF43, respectively (Fig. 3d). Furthermore, we additionally identified the transcriptional regulator PROX1 whose expression is positively correlated with cell numbers and with expression of the major intestinal stem cell marker LGR5 (Fig. 3e). This finding was validated by RNA-FISH in CRC spheroids where a low-abundant PROX1^+^ cell population was identified (Fig. 3f). We conclude that image analysis and deconvolution of pheno-seq data provides information about gene expression patterns at the single cell level even without acquiring additional single cell expression profiles.

### Linking proliferative capacity to a low-abundant stem cell subtype in CRC

**Figure 4:**
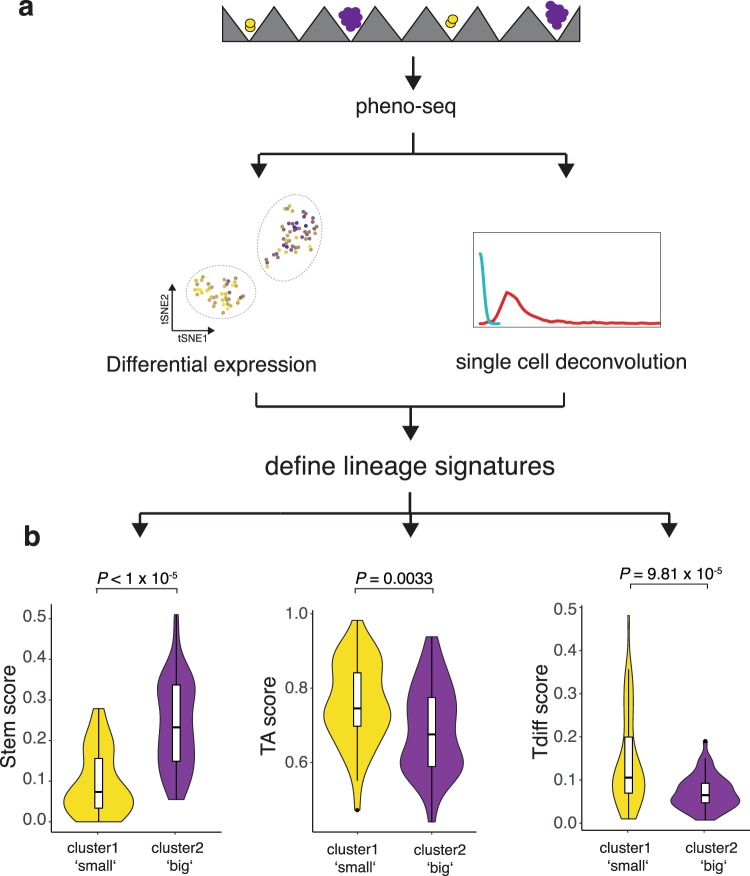
Scoring of pheno-seq data for subtype-specific signatures links long-term proliferative capacity to a stem-like subtype in CRC. (**a**) Strategy to define lineage-specific expression signatures. Stem: PROX1 correlated genes (top 20); Transit-amplifying (TA): Ribosomal genes (n = 24); Terminally differentiated (Tdiff): TFF3 correlated genes (top 20). (**b**) Violin Plots showing (cluster-specific) pheno-seq expression profiles scored for subtype signatures (see Methods). Violin-plot center-line: median; box limits: first and third quartile; whiskers: ±1.5 IQR). Indicated P-value calculated from unpaired two-tailed Students t-test.

We next aimed to directly link specific intestinal lineage subtypes to their functional proliferative phenotype. Using markers obtained from both differential expression analysis and single cell deconvolution, we defined expression programs that are specifically associated with one of the three expected and functionally different stem (long-term proliferating), transit amplifying (TA) and terminally differentiated (Tdiff, postmitotic) subtypes^4^ (Fig. 4a). We defined stem and Tdiff gene expression programs as the averaged expression of the (top 20) genes most highly correlated with PROX1 or TFF3, respectively. Grün *et al*. could link expression of ribosomal genes to a TA compartment in intestinal organoids^47^. In line with these observations, we identified a high number of ribosomal genes by pheno-seq deconvolution (GO_RIBOSOME, FDR q-value 3.53 × 10^−15^). We therefore defined a ribosomal gene signature as TA expression program (n = 24 genes).

To approximate subtype abundances in individual spheroids, we scored pheno-seq expression profiles for stem, TA and Tdiff signatures and compared scores between detected pheno-seq clusters that are associated with functional differences in proliferative capacity. Notably, whereas the ‘big’-phenotype cluster exhibited higher scores for the stem-signature, the ‘small’-phenotype cluster showed higher scores for both TA and Tdiff-signatures (Fig. 4b). These results indicate that TA and Tdiff cells, but not stem-like cells, are highly abundant in spheroids with limited proliferative capacity and that a low-abundant stem-like subtype exclusively confers long-term proliferative capacity.

## Discussion

Patient-derived 3D cell culture systems are emerging as an important approach for clinical decision making^7^. Accordingly, there is increasing need to understand the heterogeneity of functional oncogenic phenotypes in cancer. Here, we introduce pheno-seq as a novel approach to directly combine imaging and next generation sequencing at high-throughput to explain clonal heterogeneity at the morphological and molecular level.

Our method represents a complementary approach to scRNA-seq of cell suspensions, which is currently the primary method to identify cellular subpopulations: (i) Pheno-seq directly and quantitatively links heterogeneous spheroid morphologies to underlying gene expression in a single experiment. (ii) No further histological preparations are required since 3D phenotypes of whole spheroids formed by living cells are evaluated. This is critical to cover the whole spectrum of transcripts that derive from one clonal spheroid. (iii) Pheno-seq reduces dissociation biases and enables a higher transcriptome coverage per sample due to the higher cell number input. This can be advantageous to identify marker genes that are missed by scRNA-seq. (iv) Using plates or a recent ICELL8 protocol^48^, pheno-seq can be also used to analyze full-length RNA transcripts. Notably, plate-based approaches still require the manual isolation of spheroids which significantly reduces the throughput but might enable custom imaging protocols for higher resolution microscopy. (v) By integrating scRNA-seq data and applying deconvolution approaches, the resolution of the gene expression profiles can be increased to the single cell level.

As a proof-of-concept we established pheno-seq with the MCF10CA breast cancer model. We revealed epithelial and estrogen-responsive genes that define an *in-situ* like ‘round’ phenotype. Conversely, the expression of this signature decreases with more invasive (aberrant) growth behavior with simultaneous upregulation of EMT related genes.

Next, the utility of pheno-seq for analyzing functional cancer cell heterogeneity was demonstrated in a more complex model of CRC where the number of functionally distinct subtypes exceeds the number of observable phenotypes. Our results show that CRC spheroid cultures do not solely contain cancer stem cells (CSCs) but exhibit a surprisingly high degree of differentiation. In addition, the results strongly indicate that CRC ‘sphere-assays’ enable the measurement of self-renewing capacity of a distinct PROX1^+^ CSC subtype and that more differentiated subtypes have limited potential to self-renew *in-vitro*^1^. Interestingly, PROX1 is normally expressed in the intestinal enteroendocrine lineage^49^. However, two studies based on mouse tumor models suggest a role for PROX1 in cancer stem cell maintenance and metastatic outgrowth^50,51^.

We expect that the combination of functional single cell growth assay in 3D cultures with combined image and gene expression profiling will be widely applied in cancer biology, ranging from primary^8^ to circulating tumor cells (CTCs)^52^. Furthermore, the application of pheno-seq is not restricted to cancer models but could be also a valuable approach to understand non-synchronized developmental processes^6^. We believe that pheno-seq becomes even more powerful with increasing resolution and content of imaging, employing enhanced 3D-image acquisition, integrated staining by IF or live-dyes, and time-lapse microscopy, respectively. Pheno-seq can also be easily extended to other low-input, next-generation sequencing modalities such as chromatin accessibility sequencing^53^ or applied to pooled-screening approaches^54^. Thus, pheno-seq provides an additional perspective to study functional tumor cell heterogeneity in a variety of biological and clinical applications.

## Methods

### Breast cancer model MCF10CA

#### Cell culture

For 2D cell culture, the cell line MCF10CA1d clone 1 (acquired from The Barbara Ann Karmanos Cancer Institute), a transformed derivative of the MCF10A 3D-culture model for acinar morphogenesis of the mammary gland, was routinely passaged in 25 cm^2^ culture flasks. Cells were cultured in growth medium consisting of DMEM/F12 medium supplemented with 5% horse serum, 10 µg/ml Insulin, 20 ng/ml EGF, 0.5 mg/ml hydrocortisone and 100 ng/ml Cholera toxin. Cells were passaged at approximately 80% confluency with 0.05% Trypsin. The cell line was authenticated using a Multiplex human Cell line Authentication test (http://www.multiplexion.de/).

For 3D ‘on top’ assays, cells were cultured in assay medium (growth medium with only 2% horse serum and 5 ng/ml EGF) in 24-well cell culture plates. As a basement membrane surrogate, a bed of laminin-rich hydrogel (Matrigel^®^, Corning) was generated by adding 70 µm cold Matrigel into the center of pre-wetted wells. The Matrigel bed was then dried for 20 min at 37 °C. For single-cell seeding, 2D cultures were dissociated into single-cell suspensions, washed once in assay medium, passed through a 35 µm strainer and counted. Subsequently, 4000 cells were seeded per well in 400 µl assay medium with 5% Matrigel by adding cell suspensions in a 45^o^ angle to the wall of the well, which resulted in uniform distribution of single-cells throughout the well. Medium was replaced every 3 days and cells cultured for up to 12 days. All scRNA-seq and pheno-seq experiments were carried out after 5 days in 3D-culture.

#### Spheroid recovery from hydrogel

After MCF10CA cells were cultured in 3D for 5 days, medium was removed from wells and 500 µl filtered and pre-warmed Dispase (Sigma) was added. The hydrogel-matrix was detached from wells by scratching over the well bottom with a 1000 µl pipette tip and the whole Dispase-Matrigel suspension was carefully resuspended five times. Afterwards, spheroids were incubated at 37 °C for 7 min. Spheroids were then transferred to a 15 ml falcon and 5 ml assay medium was added and resuspended slowly with a 5 ml pipette. Subsequently, spheroids were spun down (300 g, 3 min) and resuspended in DMEM. We do not recommend using PBS due to perturbation of spheroid morphology. In general, this procedure resulted in approximately 2000 isolated spheroids per well.

#### Spheroid isolation and dissociation to single-cell suspensions

In order to isolate and classify individual MCF10CA spheroids prior to dissociation, suspensions were diluted to 100 spheroids per ml in assay medium and distributed into GravityTRAP^TM^ ultra-low attachment 96-well plates (PerkinElmer, 10 µl per well). Plates were centrifuged for 2 min at 250 g. The V-shaped wells with 1 mm diameter flat-bottom enabled efficient classification (round vs. aberrant) of spheroids with 10x or 20x objectives of an inverted brightfield microscope. After 40 spheroids had been isolated and identified for each class (~30–45 min), 50 µl Accumax (Sigma) was added to each well followed by an incubation of 10 min at 37 °C. To stimulate dissociation, shear forces were applied by resuspending wells of one class with a 200 µl pipette without changing the tip. After a second incubation of 5 min at 37 °C, wells of one class were pooled in 1.5 ml microcentrifuge tubes, spun down at 300 g for 3 min and resuspended in either assay medium or DMEM/F12.

#### Reseeding assay

For independent reseeding of round and aberrant 3D phenotypes, 30–40 spheroids of one class were isolated, dissociated and pooled as described above. A 10 µl Matrigel bed was prepared during dissociation in 15 µ angiogenesis slides (Ibidi). After centrifugation, cells were resuspended in 50 µl assay medium (+5% Matrigel) and added to pre-treated angiogenesis slides. Medium was replaced every 3 days and cells were cultured for up to 6 days.

#### MCF10CA single-cell capture, mRNA library preparation and sequencing

For MCF10CA single-cell RNA sequencing experiments, spheroids were dissociated as described above and resuspended in DMEM/F12 medium. Capture, full-length cDNA synthesis and amplification was performed on the C1 Single-Cell Auto Prep system for mRNA Seq (Fluidigm) using the IFC for up to 96 cells (medium size 10–17 µm). Cells at a concentration of 350 cells/µl were mixed with C1 Cell Suspension Reagent (Fluidigm) at a ratio of 4:1 immediately before loading on the IFC. Single-cell capture was assessed with an inverted brightfield microscope. Workflow and reagents for single-cell RNA extraction, reverse transcription (RT) and mRNA amplification (18 cycles) were used as described in the SMARTer Ultra Low RNA Kit (for Fluidigm C1). Sequencing libraries were generated with the Nextera XT kit (Illumina) according to an adapted Fluidigm protocol. Concentration and quality of cDNA and sequencing libraries was assessed by a fluorometer (Qubit) and by electrophoresis (Agilent Bioanalyzer high sensitivity DNA chips). Libraries of up to 24 cells were pooled and sequenced as 1 × 50-bp reads on an Illumina HiSeq 2000 machine.

#### Full length pheno-seq workflow, library preparation and sequencing

For full-length pheno-seq, suspensions were diluted to 500 spheroids per ml in DMEM and 2 µl was carefully dispensed to the wall of the well of GravityTRAP^TM^ 96-well plates followed by vertical tapping of the plate. Wells with single spheroids were then microscopically classified. For RNA extraction with the Arcturus PicoPure kit (ThermoFisher), 50 µl extraction buffer was directly added to 96-wells, incubated for 2 min at RT and then transferred to 1.5 ml LoBind microcentrifuge tubes (Eppendorf). RNA was isolated as described in the PicoPure Kit (Appendix B and Section 4B.2) including on-column DNase digestion (Appendix A, RNase-Free DNAse Set, Qiagen). RNA was eluted in Nuclease-free water (~10 µl) and used as input for full-length cDNA synthesis and amplification (16 cycles) by the SMART-Seq^®^ v4 Ultra Low Input RNA Kit for sequencing (TakaraBio). Sequencing libraries were generated with the Nextera XT kit (Illumina) as described in the SMART-Seq^®^ v4 protocol. Concentration and quality of cDNA and sequencing libraries was assessed by a fluorometer (Qubit) and by electrophoresis (Agilent Bioanalyzer high sensitivity DNA chips). Ten libraries were pooled and sequenced as 1 × 50-bp reads on an Illumina HiSeq 2000 machine.

#### MCF10CA high-throughput pheno-seq workflow, library preparation and sequencing

The following section describes the primary high-throughput pheno-seq workflow, including staining, cellular fixation, dispensing, cDNA amplification and NGS library preparation. For detailed description of microscopy and image analysis see section ‘Microscopy and image analysis’ and supplementary information.

For high-throughput (HT-)pheno-seq, we adapted and improved the nanowell-based Wafergen iCELL8 scRNA-seq system, that integrates imaging and gene expression profiling of big samples of up to 100 µm^55^. First, spheroids were stained 3 hours with 10 µM CellTracker^TM^ Red CMTPX dye and 1 µg/ml Hoechst 33258 (ThermoFisher). Afterwards, spheroids of six wells were recovered as described above and washed once with 7 ml DMEM (Life Technologies). Only three wells were pooled per 15 ml falcon tube for centrifugation. The reversible cross-linker dithio-bis(succinimidyl propionate) (DSP) was prepared for cellular fixation as previously described^32^ and directly filtered through a 10 µm strainer. Spheroids were resuspended in 400 µl DSP and incubated for 30 min at room temperature. After fixation, spheroids were washed two times with cold PBS (centrifugation at 650 g and 500 g, 3 min, 4 °C) and then resuspended in 650 µl cold PBS with 1x second diluent (for iCELL8) and 0.4 U/µl recombinant RNase Inhibitor (TakaraBio). Spheroids were dispensed into a barcoded 5184-nanowell chip with the iCELL8 Single-Cell System (TakaraBio) as described in the Rapid Development Protocol (in-chip RT-PCR amplification). As a control, we first dispensed, imaged and processed one chip without cellular fixation using the default settings, the standard microscope and the provided CellSelect^TM^ software.

For improved HT-pheno-seq we applied the following modifications: Between the three dispensing intervals, wells in the 384-well source plate were stirred with a 200 µl pipette tip just before intake of suspensions with the dispensing heads in order to minimize settling of spheroids and to enable even distribution in nanowells. Similar to the standard single-cell protocol, the iCELL8 chip was tightly sealed with a strongly adhesive imaging foil (TakaraBio). Instead of spinning cells to the bottom, spheroids were centrifuged upside-down to the foil (700 g, 5 min, 4 °C, decelerated break) in order to reduce the working distance and to avoid light reflections deep inside the well during imaging.

To further enhance imaging resolution, we used an inverted confocal laser-scanning microscope (Leica SP8) with a 10x objective (2 × 2 wells per field of view) instead of the standard and system-integrated fluorescence wide-field microscope.

Afterwards, spheroids were centrifuged to the bottom (700 g, 5 min, 4 °C) and chips were frozen at −80 °C. A ‘filter file’ was used to dispense reagents only in selected nanowells as described in the Rapid Development Protocol (TakaraBio), with the exception that we adjusted the amount of Triton-X100 to a final well concentration of 1% for spheroids lysis. The timing of spheroid recovery and consequently the maximum spheroid size (that correlates with the number of cells per spheroid/well) should not exceed 100 µm as this might negatively influence RT efficiency. In addition, lysis reagents, concentration and duration might have to be adjusted for different culture models.

After in-chip reverse transcription and cDNA amplification (18 cycles), barcoded cDNA was pooled and processed to 3′-end sequencing libraries by the Nextera XT kit (Illumina) with specific adaptions described in the Rapid Development Protocol. Concentration and quality of cDNA and sequencing libraries was assessed by a fluorometer (Qubit) and by electrophoresis (Agilent Bioanalyzer high sensitivity DNA chips). Improved HT-pheno-seq paired-end iCELL8 libraries (21 + 70) were sequenced on an Illumina NextSeq 500 machine in high-output mode. The ‘bottom control’ chip without improved imaging was sequenced on a HiSeq 2000 machine with similar settings. However, this control was only used to assess library quality and not for further downstream analysis.

### Colon TICs spheroids

#### Cell culture

Primary patient-derived colon tumor spheroid cultures were established as described previously^4^. Primary human colon cancer samples were obtained from Heidelberg University Hospital in accordance with the declaration of Helsinki. Informed consent on tissue collection was received from each patient, as approved by the Review Board of the Ethics Committee of the University Clinic Heidelberg. The culture used in this study was derived from a liver metastasis. Cells were cultured in 75 cm^2^ ultra-low attachment flasks in advanced D-MEM/F-12 medium supplemented with Glucose (0.6%), 2 mM L-glutamine, 4 μg/ml heparin, 5 mM HEPES, 4 mg/ml BSA, 10 ng/ml FGF basic and 20 ng/ml EGF. Growth factors were added every 4 days and medium was exchanged every 4–8 days. For dissociation to single-cell suspensions, spheroid cultures were centrifuged for 5 min at 900 rpm and resuspended in 2–4 ml 0.25% Trypsin. To stimulate dissociation, shear forces were applied with a 1000 µl pipette every 5 min for 20 min in total. Subsequently, 4–8 ml stop solution (PBS with 20% heat inactivated and sterile filtered fetal bovine serum) was added and cells were centrifuged for 5 min at 900 rpm. For passaging, cells were then resuspended in medium, passed through a 40 µm strainer and counted.

#### Reseeding assay

To isolate, dissociate and reseed cells from big (70–100 µm) and small (20–40 µm) spheroids independently, we cultured colon spheroids for 10 days and performed a stepwise size exclusion by (reverse-) filtering with standard 100 µm, 70 µm, 40 µm and 20 µm cell strainers, respectively. Spheroids were dissociated to single-cell suspension as described above but passed through a 15 µm cell strainer and counted. Afterwards, 50,000 cells were seeded in 60 mm Ultra Low Attachment Culture Dishes (Corning). Growth factors were added every 4 days and cells cultured for 10 days. Culture dishes were shaken every day to avoid clustering of spheroids.

#### Single-cell culture and pheno-seq of colon tumor spheroids

For single-cell cultures of colon tumor cells, spheroids were dissociated to single-cell suspensions, passed through a 15 µm cell strainer and counted. Cells were cultured in Aggrewell 400 6-Well plates (StemCell Technologies) in which each well contains a standardized array of around 7000 inverse pyramidal shaped microwells with a size of 400 µm. For seeding, wells were pre-treated according to the manufacturer’s instructions, washed once with PBS and once with medium. Subsequently, 3500 cells in 3 ml medium were added in a 45^o^ angle to the wall of the well, which resulted in uniform distribution of single-cells in microwells after settling. Growth factors were added every 4 days and cells were cultured for 10 days, resulting in 300–400 spheroids (>20 µm) per 6-well. Spheroids from 4–6 plates (24–36 wells, 168,000–252,000 microwells) were harvested, pooled and washed once with FluoroBrite DMEM (Life Technologies, 900 rpm for 5 min).

HT-pheno-seq was performed as described for MCF10CA spheroids above, but with following modifications: In contrast to MCF10CA spheroids, colon spheroids did not require DSP fixation because spheroid recovery does not involve contact loss from reconstituted basement membrane (Matrigel). To minimize disassembly of spheroids during processing, cells were resuspended and dispensed in FluoroBrite DMEM instead of PBS.

### Microscopy and image analysis

#### Image processing and analysis

Generally, acquired microscopy images were processed and analyzed using KNIME Image Processing (https://www.knime.com/community/image-processing, Version 3.2.1), ImageJ (https://imagej.nih.gov/ij/), R (Version 3.3.1)/R studio (https://www.rstudio.com/) and/or Graph Pad Prism 7 (https://www.graphpad.com /scientific-software/prism/). Generally, the ggplot2 package implemented in R and Graph Pad Prism 7 were used for data visualization and the PhenoSelect webtool design is based on the shiny package (https://shiny.rstudio.com). More detailed information on microscopy and image analysis can be found in the supplementary information file and in associated KNIME workflows deposited in the pheno-seq github repository (https://github.com/eilslabs/pheno-seq).

#### HT-pheno-seq microscopy and image processing/analysis

The following section describes the basic microscopy setup and imaging parameters for HT-pheno-seq as well as major steps for image processing and analysis. For further details, see supplementary methods.

For inverted imaging, 5184-nanowell iCELL8 chips were fixed on a metallic Chip Spinner (TakaraBio) with adhesive tape and placed into a standard plate holder. All wells were imaged upside-down automatically using an inverted Leica SP8 confocal microscope system. We used a 10×/0.30 air objective (Leica HC PL FLUOTAR) but images were acquired with 0.9x digital zoom to span 4 wells per field of view. Excitation was set to 405 and 552 nm and emission filter were set to receive signals between 415–485 nm (Hoechst) and 555–625 nm (CellTracker Red), respectively. A pre-defined HCS A template of the LAS X microscope software (Leica) was used for the grid design matching the chip dimensions. One image contained 512 × 512 pixels, with 2.53 μm pixel size. Scanning of one chip with these settings took approximately 30 minutes, resulting in 2 × 1296 images.

Images were automatically processed using KNIME/ImageJ for assigning images to their correct well positions, image cropping, spheroid detection and segmentation as well as feature extraction and quantification. The web-based shiny app ‘PhenoSelect’ was used for final selection of wells and for interactive analysis (see supplement for further details).

#### Immunofluorescence

MCF10CA cells cultured in 3D were prepared for immunofluorescence staining as described previously^28^. Briefly, cells were fixed in 24-wells with 2% Formaldehyde solution (Methanol-free, ThermoFisher) for 20 min at RT and washed twice with PBS. Cells were permeabilized with PBS + 0.5% TritonX-100 (Sigma) for 10 min and washed three times with PBS + 75 mg/ml Glycine (pH = 7.4, Sigma). Unspecific binding sites were blocked for 1 hour at RT with 10% goat serum in IF-wash solution (PBS + 5 mg/ml NaN_3_, 10 mg/ml bovine serum albumin, 2% TritonX-100 and 0.4% Tween20, pH = 7.4, Sigma). Afterwards, primary antibodies in blocking solution were added and incubated at 4 °C overnight. The next day, cells were washed 3x with IF-wash and then incubated with fluorescently labeled secondary antibodies in blocking solution for 1 hour at RT if primary antibodies were unlabeled. Subsequently, cells were washed 3x with IF-wash and 2x with PBS and then incubated in PBS + 1 µg/ml Hoechst for 20 min at RT. Cells were again washed with PBS, removed from the surface and transferred into 8-well Nunc™ Lab-Tek™ Chamber Slides (ThermoFisher) for improved fluorescence detection. The following antibodies were used in this study: Rabbit anti-Vimentin antibody Alexa Fluor^®^ 594 (1:100, EPR3776, abcam), mouse anti-β-Actin antibody (1:200, 8H10D10, Cell Signaling), Mouse anti-Cytokeratin 15 antibody (1:50, LHK15, ThermoFisher), Goat anti-mouse Alexa Fluor^®^ 594 (1:200, Cell Signaling). 3 × 3 images per well (20 Z-stacks per position) were acquired automatically on a Zeiss LSM780 Axio Observer confocal microscope equipped with a 10x/0.3 air objective (Zeiss EC PLAN-NEOFLUAR) using a custom Zeiss VBA macro. Beside brightfield images, lasers and filters were set to measure fluorescence emitted from Hoechst (DNA) and from Alexa Fluor^®^ 594-labeled antibodies. Images were analyzed using a custom KNIME workflow in which protein abundances per classified spheroid were defined as mean pixel intensity of the fluorescence signal emitted from labeled antibodies.

Z-stacks of whole mount stained MCF10CA spheroids were first merged by average intensity projection and a mask for single spheroids was created based on the Hoechst signal. Therefore, images were smoothed by Gaussian convolution (sigma = 2) and thresholded by Otsu’s method. Labels were assigned to objects (spheroids) by connected component analysis and objects smaller than 300 and bigger than 800,000 pixels were filtered out to remove noise as well as segmentation artifacts. To compare expression of antibody targets between round and aberrant 3D phenotypes, single objects (spheroids) were manually classified as ‘round’ or ‘aberrant’ based on brightfield images. Protein abundances per spheroid were defined as mean pixel intensity of the fluorescence signal emitted from labeled antibodies.

#### RNA FISH

For histological preparation, ‘big’ (70–100 µm) and ‘small’ (70–100 µm) colon tumor spheroids derived from single-cells were isolated with (reverse-) filtering as described above. This step was added for histological preparation in order to distinguish between small spheroids and big spheroids that were sliced in peripheral regions. Spheroids were then fixed with 4% Formaldehyde solution for 20 min at 4 °C, washed twice with PBS and incubated in 30% sucrose at 4 °C overnight. The next day, spheroids were embedded in Neg-50™ and frozen in the gaseous phase of liquid nitrogen.

For both cultures, sectioning was performed at −20 °C on a cryostat (Leica) and 10 µm slices were mounted on Superfrost Plus slides (ThermoFisher). Embedded specimens and cryosections were stored at −80 °C until further use.

For highly sensitive RNA fluorescence *in-situ* hybridization (RNA-FISH), we employed the RNAscope^®^ Fluorescent Multiplex Assay 2.0 (ACDbio). Cryosections were processed as described in the ‘Sample Preparation Technical Note for Fixed Frozen Tissue’ and the ‘Fluorescent Multiplex Kit User Manual PART 2′. Briefly, cryosections were pretreated with Protease IV (ACDbio) for 15 min at RT. Afterwards, transcript-specific probes were hybridized at 40 °C for 90 min followed by stepwise hybridization of probes for signal amplification and fluorescent detection (Amp-1-FL – Amp-4-FL). Up to three transcripts were labeled by Alexa488, Atto550 and Atto647 fluorescent dyes. Following mRNA targeting probes were used: MYC (Atto550, #311761-C2), CD44 (Atto647, #311271-C3), TFF3 (Alexa488, #403101), PROX1 (Atto550, #530241-C2). Finally, cryosections were counterstained with DAPI, mounted in SlowFade^TM^ Gold Antifade solution (ThermoFisher) and stored at 4 °C until further use.

RNA-FISH images were acquired on a Leica SP8 confocal laser-scanning microscope equipped with a 40x/1.30 oil objective (Leica HC APO CS2). Images of individual spheroids at 1024 × 1024 pixel resolution were generated semi-automatically using the ‘Mark and Find’ option in the Leica SP8 acquisition software. To cover the whole 10 µm cryosection height, a Z-range of 20 µm was acquired by 15 stacks (1.43 µm distance between frames). Lasers and filters were set to match fluorescent properties of DAPI and abovementioned dyes. For analysis of RNA-FISH imaging data we used a custom KNIME workflow in which we defined the relative transcript expression per spheroid as quantified pixel percentage that exceeds a calculated background threshold per spheroid.

Z-stacks acquired from histology slides were merged using maximum intensity projection and a mask for single spheroids was created using the DAPI signal. Briefly, acquired DAPI signals were smoothed by applying Gaussian convolution (sigma = 5) and a maximum filter with a radius of 12 pixels, resulting in individual masks for all spheroids within an image. Only the biggest object/spheroid was used for analysis if two or more objects were present in one image. To quantify transcript abundances (measured as fluorescence intensities derived from specifically labeled probes) we first accounted for background noise by fitting two local maxima, j and k, to the pixel intensity histogram of each spheroid using the ‘intermodes’ method in KNIME. Based on the determined probe-specific pixel intensity threshold between two maxima calculated as (j + k)/2, we defined the relative transcript expression per spheroid as quantified pixel percentage that exceeds this threshold per object.

### Sequencing data analysis

#### Pre-processing of RNA-seq data and library quality control

An automated in-house workflow was established for pheno-seq and Fluidigm C1 scRNA-seq data pre-processing. Briefly, short read quality was evaluated using FastQC. For iCELL8 libraries, barcodes from the first 21 bp read were assigned to the well of origin with the Je demultiplexing suite^56^. Cutadapt was used to trim remaining primer sequences, Poly-A/T tails and low-quality ends (<25). In addition, since NextSeq (Illumina) encodes undetected base as incorrect ‘G’ with high quality, Cutadapt’s ‘—nextseq-trim’ option was used for correct quality trimming. Trimmed reads were mapped to the reference genome hs37d5 (1000 genomes project) using STAR aligner. Mapped BAM files were quantified using featureCounts with gencode v19 as reference annotation.

RNA-seq libraries that did not match the following criteria were filtered out: MCF10CA scRNA-seq: (i) > 300,000 reads, (ii) > 3000 detected genes (i.e. > 0 read count), (iii) < 10% mitochondrial reads; MCF10CA pheno-seq: (i) > 100,000 reads, (ii) > 2000 detected genes, (iii) < 15% mitochondrial reads; Colon spheroid pheno-seq: (i) > 200,000 reads, (ii) > 3000 detected genes, (iii) < 15% mitochondrial reads.

In order to compare the performance of scRNA-seq and pheno-seq methods in detecting genes, MCF10CA sequencing libraries were downsampled to 100,000 reads by a custom R script.

Wells/Spheroids with imaging artifacts (e.g. segmentation errors) were removed if detected during combined downstream analysis.

#### PAGODA/SCDE subpopulation and differential expression analysis

To identify expression signatures that separate distinct cellular subpopulations, we analyzed transcriptional heterogeneity by pathway and gene set overdispersion analysis (PAGODA/SCDE-package^33^). First, genes with less than 10 mapped reads in the whole dataset were not considered for further analysis. Next, PAGODA constructs error models for individual cells using a binominal/Poisson mixture model, thereby controlling for technical aspects of variability, like effective sequencing depth, drop-out rate and amplification noise. For K-nearest neighbor error modelling, k was set to 30 (except for the full-length pheno-Seq dataset: k = 3), and the minimum number of reads required to be considered non-failed was set to 2. Afterwards, PAGODA performs weighted principal component analysis (wPCA) on annotated and *de-novo* identified gene sets in order to identify those that exhibit statistically significant variability. Generally, the scores for the first principal component are presented if not stated otherwise. Annotated hallmark (H) and gene ontology (GO_C5) gene sets were derived from the Molecular Signature Database (MSigDB). *De-novo* gene sets were identified by hierarchical clustering (Ward method; dendrogram was cut into 150 clusters). Pathway overdispersion was calculated as Z-score relative to the genome-wide model and corrected Z-scores (cZ) were computed using multiple hypothesis testing using the Holm procedure. Hierarchical clustering is then performed on the top significant aspects of heterogeneity and redundant aspects of heterogeneity were grouped with a similarity threshold of 0.7. Up to 10 top significant aspects were used for visualization. In addition, 2D t-SNE maps^57^ were generated based on PAGODA’s weighted Pearson correlation distances. Finally, the following confounding expression signatures (e.g. technical aspect or cell cycle influence) were removed using the ‘pagoda.substract.aspect’ function:

1. For all datasets we corrected for the influence of gene coverage (estimated as a number of genes with non-zero magnitude per cell)
2. MCF10CA scRNA-seq: GO_REGULATION_OF_CELL_CYCLE and HALLMARK _G2M_CHECKPOINT;
3. MCF10CA HT-pheno-seq: GO_ NUCLEOSIDE_MONOPHOSPHATE_ METABOLIC _PROCESS, GO_MITOCHONDRIAL_ENVELOPE, GO_STRUCTURAL _MOLECULE _ACTIVITY, GO_ HOMEOSTATIC_PROCESS and associated *de-novo* identified gene sets.

Differentially expressed genes (MCF10CA: fold change >1.3; adjusted p-value < 0.1; CRC spheroids: fold change >1.5; adjusted p-value < 0.05) between detected subpopulations that refer to observed visual phenotypes (k-means clustering, k = 2) were identified by the SCDE-package^37^.

#### *In-silico* reconstruction of synthetic pheno-seq expression profiles from single-cell data

Synthetic spheroid expression profiles were reconstructed from scRNA-seq data by randomly dividing cells either derived from round and aberrant phenotypes in four groups each in four independent randomizations. Read counts for each gene were then averaged over each group, resulting in eight synthetic spheroid profiles (4 round and 4 aberrant) that were then analyzed by PAGODA similar to the full-length pheno-seq dataset.

#### Deconvolution of the CRC spheroid dataset by maximum likelihood inference

In order to infer heterogeneous regulatory states informative for single cell expression by deconvolution, we adapted a maximum likelihood inference approach initially developed to identify cell-to-cell heterogeneities from random 10-cell samples^45^ (Stochastic Profiling, Fig. 3a). The adapted algorithm uses the estimated cell numbers per spheroid to fit two log-normal distributions (LN-LN model) to given ‘mixed-n’ datasets in order to identify genes with bimodal expression pattern at the single-cell level (Stochastic Profiling). Here, we allowed each sample to consist of different numbers of cells (implemented in the R package stochprofML version 2.0: https://github.com/fuchslab/stochprofML).

The algorithm assumes that the expression of a spheroid linearly scales with its cell number. We approximated absolute counts per spheroid by using estimated cell numbers derived from light sheet microscopy and image analysis and normalized pheno-seq data as follows: First, counts per spheroid were divided by the respective estimated cell number, and the minimal average mRNA count per cell was determined (2374.644). Afterwards we downsampled the whole dataset to 2300 counts per cell resulting in a perfect correlation of mRNA counts and cell numbers. (Supplementary Fig. 9b). The downsampled dataset was filtered by removing genes with less than one count per well on average over the original CRC spheroid dataset and genes with less than 5 counts in at least two wells, leaving 13,868 genes that are taken into account during the profiling procedure. To avoid problems with zeros and log-normal distributions, all zeros were transformed to 0.1.

#### Scoring of pheno-seq data for subtype-specific gene expression signatures

First, meta-signatures for predicted single cell subtypes (stem, transit amplifying and terminally differentiated) were defined as the averaged expression of the top 20 genes most highly correlated with PROX1 (stem) or TFF3 (Tdiff). The TA signature was defined as average expression of ribosomal genes identified by pheno-seq deconvolution (n = 24 genes). We used control random gene sets as a background model in order to control for technical confounders^20^. To link subtype-specific expression to pheno-seq clusters, downsampled spheroid expression profiles (see Stochastic Profiling normalization) were scored for defined signatures. Finally, signature scores for the two identified pheno-seq clusters (‘big’ and ‘small’-phenotype) were compared using unpaired two-tailed Students t-test.

#### Statistical analysis and visualization

Statistical analysis and visualization of sequencing data was done in R (Version 3.3.1) or R studio (https://www.rstudio.com/) using PAGODA/SCDE^33^, (Version 2.3), ggplot2, ComplexHeatmaps^58^, the stats package (R version 3.3.1), stochprofML (R version 3.4.1) and in Graph Pad Prism 7 (https://www.graphpad.com/scientific-software/prism/). Gene set enrichment analysis was done by computing overlaps between identified class-specific signatures and gene sets derived from the Molecular Signature Database^38^ (MSigDB, https://software.broadinstitute.org/gsea/msigdb).

## Supplementary information

Supplementary Information

## Data Availability

Raw sequencing data for MCF10CA are accessible at the European Nucleotide Archive (https://www.ebi.ac.uk/ena) under Accession Number PRJEB26737. Colon tumor spheroid raw sequencing data have been deposited at the European Genome‐Phenome Archive (http://www.ebi.ac.uk/ega/) under Accession Number EGAS00001002999. All KNIME image analysis workflows, R code for PhenoSelect and PAGODA/SCDE RNA-seq analysis as well as a download link for MCF10CA HT-pheno-seq image data with all necessary components to run the pre-processing workflow and/or the PhenoSelect web app can be found in the pheno-seq github repository (https://github.com/eilslabs/pheno-seq). Information on the automated in-house RNA-seq workflow is available upon request. The newest version of stochProfML 3.4.1 can be found under: https://github.com/fuchslab/stochprofML.
