# Supplementary material for: Pheno-seq – linking visual features and gene expression in 3D cell culture systems
Source: Sci Rep. 2019 Aug 26;9:12367. doi: 10.1038/s41598-019-48771-4 (PMC6710272; doi:10.1038/s41598-019-48771-4)
Supplement: Supplementary file 1 — Supplementary Information [file 41598_2019_48771_MOESM1_ESM.docx]

# Supplementary information

**pheno-seq – linking visual features to gene expression in 3D cell culture systems**

*Stephan M. Tirier^2,3,7*^, Jeongbin Park^1,3^, Friedrich Preußer^,2,3,4*^, Lisa Amrhein^5,6^, Zuguang Gu^3,8^, Simon Steiger^2,3^, Jan-Philipp Mallm^2,7,8^, Teresa Krieger^1,2,3^, Marcel Waschow^2,3^, Björn Eismann^2,3^, Marta Gut^9,10^, Ivo G. Gut^9,10^, Karsten Rippe^2,7^, Matthias Schlesner^3,11^, Fabian Theis^5,6^, Christiane Fuchs^5,6,12^, Claudia R. Ball^13^, Hanno Glimm^13,14^, Roland Eils^1,2,3,8,15^, Christian Conrad^1,2,3,8,†^*

**Affiliations**

^1^Digital Health Center, Berlin Institute of Health (BIH)/Charité-Universitätsmedizin Berlin, Berlin, Germany

^2^Center for Quantitative Analysis of Molecular and Cellular Biosystems (BioQuant), University of Heidelberg, Heidelberg, Germany.

^3^Division of Theoretical Bioinformatics, German Cancer Research Center (DKFZ), Heidelberg, Germany.

^4^Max Delbrück Center for Molecular Medicine, Berlin Institute for Medical Systems Biology, Berlin, Germany

^5^Helmholtz Zentrum München - German Research Center for Environmental Health, Institute of Computational Biology, Munich, Neuherberg, Germany

^6^Department of Mathematics, Technische Universität München, Munich, Germany

^7^Division of Chromatin Networks, German Cancer Research Center (DKFZ), Heidelberg, Germany.

^8^Heidelberg Center for Personalized Oncology, DKFZ-HIPO, DKFZ, Heidelberg, Germany.

^9^CNAG-CRG, Centre for Genomic Regulation, Barcelona Institute of Science and Technology, Barcelona, Spain.

^10^Universitat Pompeu Fabra, Barcelona, Spain.

^11^Bioinformatics and Omics Data Analytics, German Cancer Research Center (DKFZ), Heidelberg, Germany.

^12^Faculty of Business Administration and Economics, Bielefeld University, Bielefeld, Germany.

^13^Department of Translational Oncology, NCT Dresden, University Hospital, Carl Gustav Carus, Technische Universität Dresden, Dresden and DKFZ, Heidelberg, Germany

^14^German Cancer Consortium, Heidelberg, Germany.

^15^Health Data Science Unit, University Hospital Heidelberg, Heidelberg, Germany.

^†^Corresponding author: christian.conrad@bihealth.de

*Current address

Keywords: 3D cell culture, tumor cell heterogeneity, single cell analysis, gene expression deconvolution, spheroid image analysis

## Supplementary figures and tables


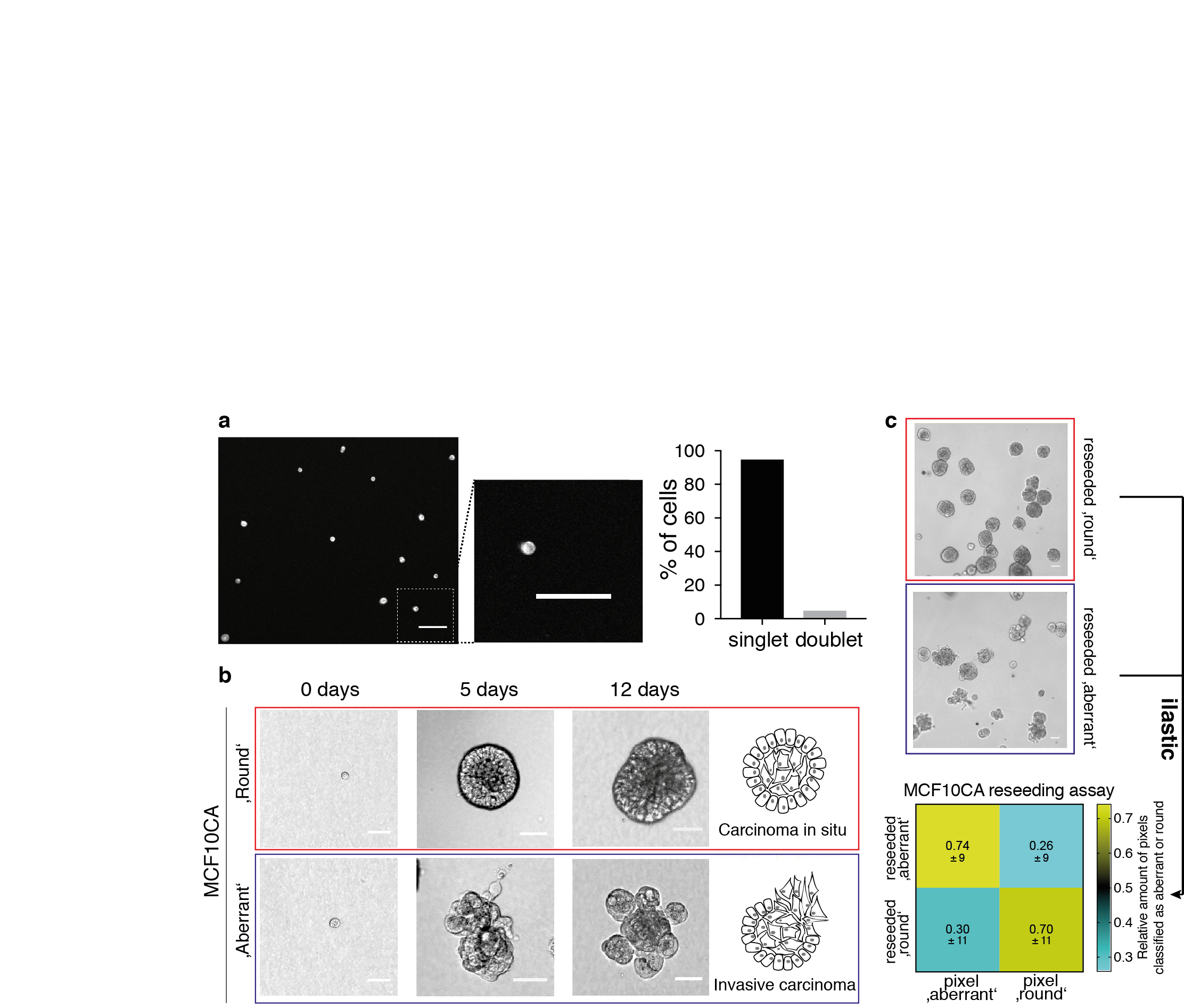


Supplementary Figure 1 | Heterogeneous 3D breast cancer model MCF10CA

(a) Single-cell seeding efficiency assessed by image analysis. Left: Example image of CellTacker Red stained and seeded cells (scale bar: 100 µm). Middle: Magnified image that corresponds to dashed box in left image. Right: Quantified cell singlets and doublets after seeding (289 objects in total). (b) Brightfield microscopy images of heterogeneous MCF10CA spheroids after 0, 5 and 12 days of culture in Matrigel, thereby reflecting histological characteristics of key steps during malignant progression of breast cancer (Brightfield, scale bar 50 µm). Red box: ‘round’ phenotype; Blue box: ‘aberrant’ phenotype. (c) Independent reseeding of isolated ‘round’ and ‘aberrant’ spheroid phenotypes and quantification after regrowth by ‘ilastic’ machine learning based on pixel classification. Lower: Spheroid classification confusion matrix. Heatmap reflecting classiﬁed pixels as aberrant or round after reseeding (four replicates, indicated are relative pixel numbers and standard error of the mean below). Upper: Example images of reseeded MCF10CA ‘round’ and ‘aberrant’ spheroids 5 days after reseeding (scale bar: 50 µm)


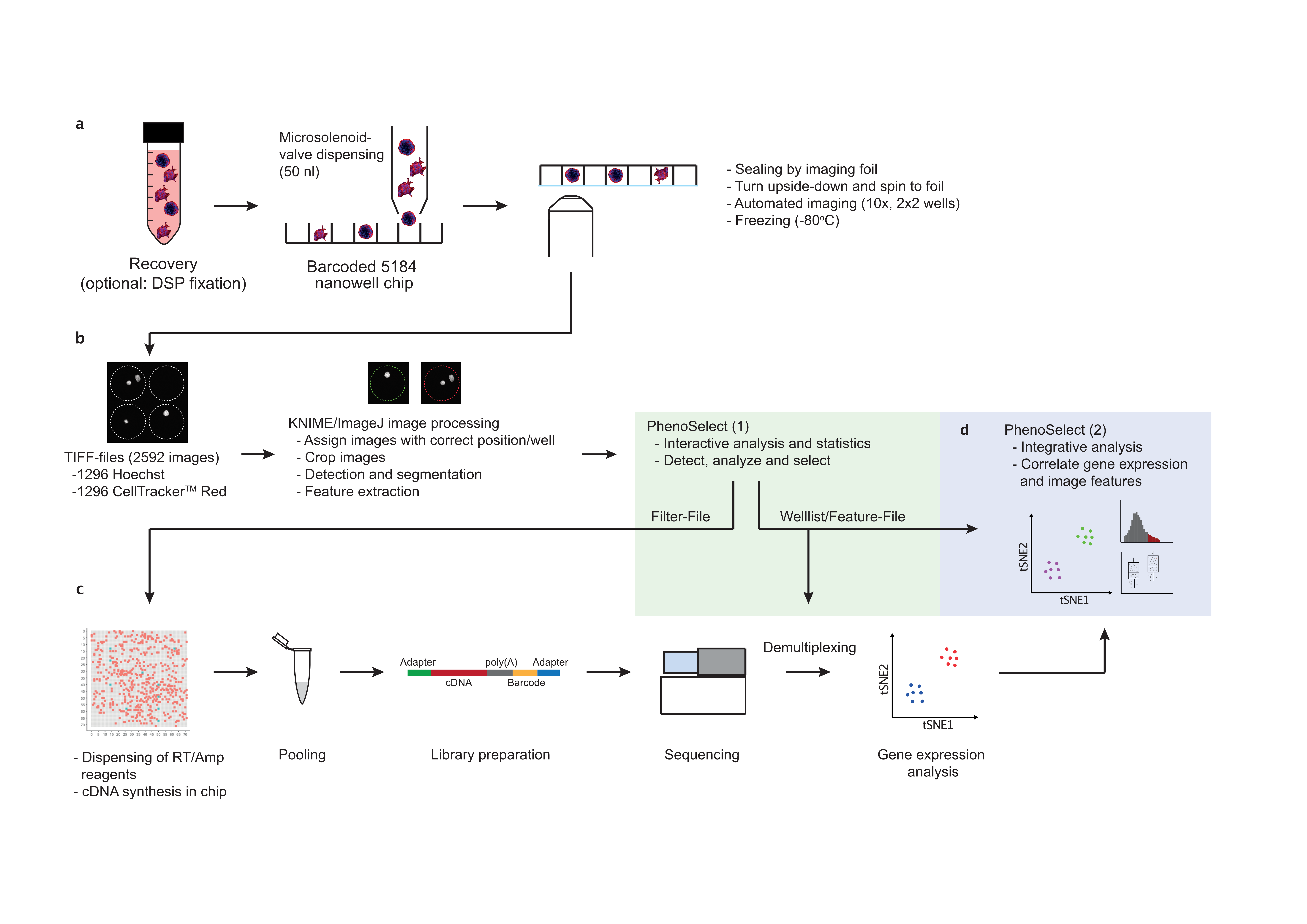


Supplementary Figure 2 | Detailed pheno-seq workflow.

**(a)** After staining and recovery (optional: DSP fixation), spheroids are distributed into a nanowell chip by a Microsolenoid-valve dispenser (50 nl per well). To improve imaging quality, spheroids are centrifuged upside-down to the foil and automatically imaged by an inverted confocal microscope. The chip is then frozen at -80^o^C. **(b)** Images are processed using a custom-made image processing pipeline in KNIME/ImageJ. A Shiny-based web-app (PhenoSelect) enables interactive analysis and selection based on quantified image features. **(c)** A filter-file generated by PhenoSelect is used to dispense RT/Amp reagents only in selected wells. cDNA generation and amplification are performed in the chip. After pooling of barcoded cDNA, 3´-library generation and next generation sequencing, resulting raw data can be de-multiplexed using internal barcodes listed in the welllist/feature-file generated with PhenoSelect. **(d)** Combined image analysis enables combined analysis of gene expression and image features.


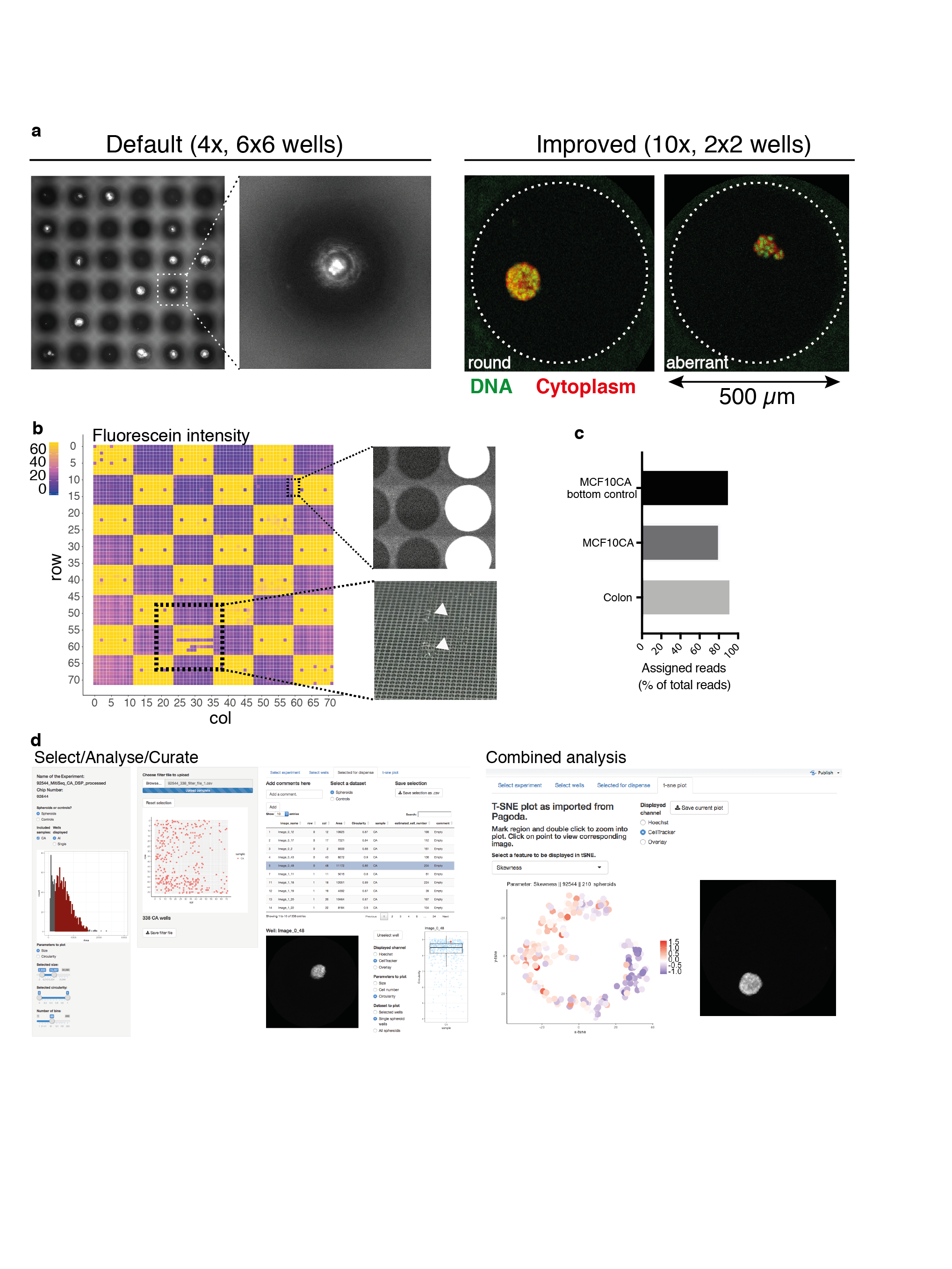


Supplementary Figure 3 | Technical adaptions and controls for pheno-seq and PhenoSelect webtool.

**(a)** Comparison of images acquired by the default microscope with 4x objective, capturing 6x6 wells per image (spheroid nuclei are stained with Hoechst dye), and higher resolution microscopy (Confocal Leica SP8) with 10x objective, capturing 2x2 wells per image (spheroids are stained with Hoechst dye and CellTracker Red CMTPX). **(b)** Leakage analysis by patterned Fluorescein dispensing. Average fluorescence intensity is plotted onto 72x72 well grid that corresponds to nanowell chip architecture (left). For better visualization, all average intensity values exceeding 77 were set to maximum in the color code scheme. Top right: Example image showing the border between wells that have been filled with PBS or PBS with Fluorescein. Lower right: Macroscopic image of nanowell surface with droplets, showing rare dispensing errors that are also reflected by absence of Fluorescein signal at the respective position. **(c)** High percentage of reads that only map to selected well barcodes excludes severe leakage of barcoded Poly-T primers upon centrifugation of spheroids to the foil.


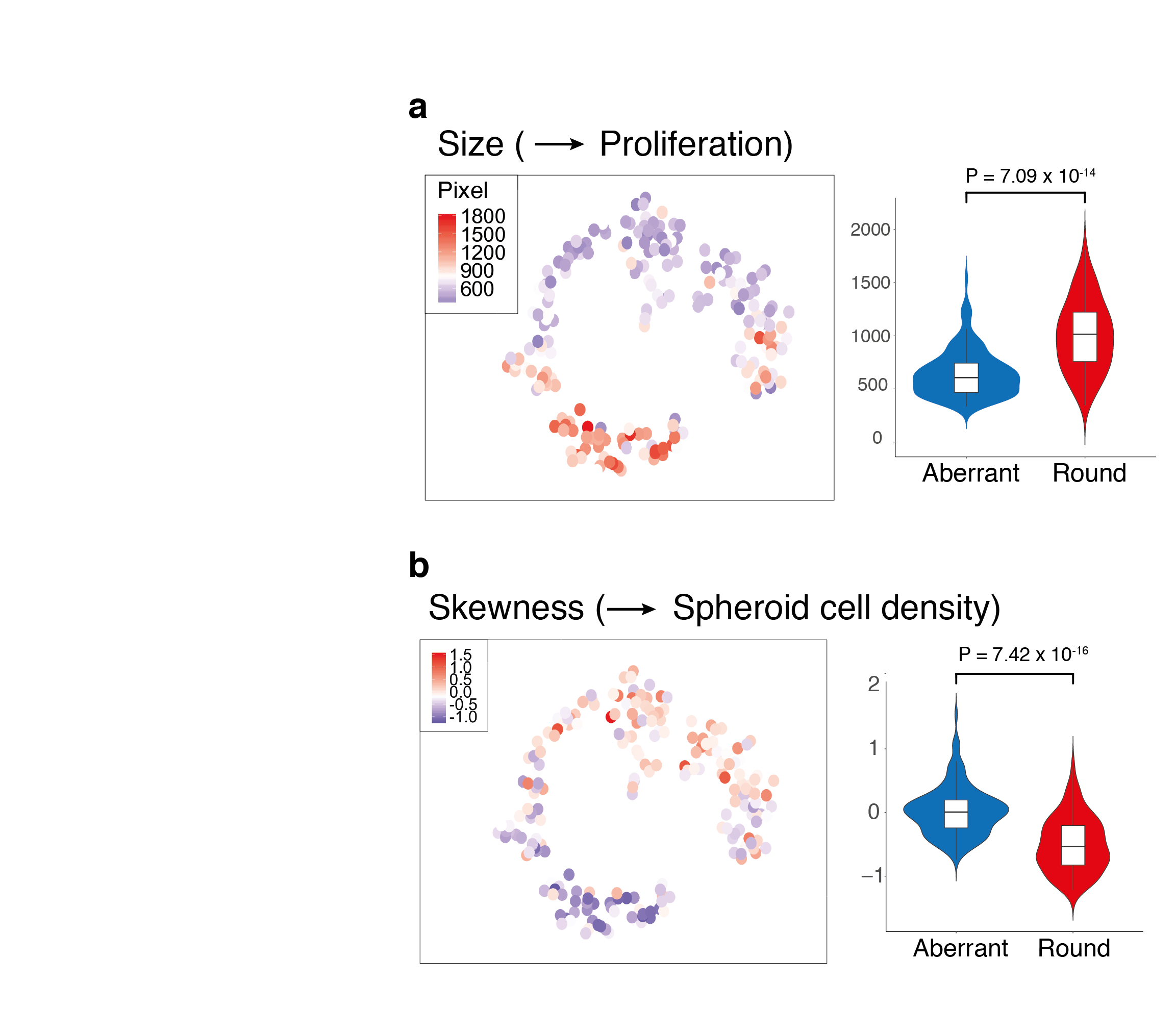


Supplementary Figure 4 | Additional image features that are linked to gene expression clusters in MCF10CA pheno-seq data

**(a and b)** PAGODA 2D tSNE embedding of MCF10CA pheno-seq dataset colored for image features ‘size’ (a) and ‘skewness’ (b) and associated Violin plots for image feature quantification per cluster (k-means clustering: k=2; violin center-line: median; box limits: first and third quartile; whiskers: ±1.5 IQR; Indicated *P*-values from unpaired two-tailed Students t-test). Image feature associations can be interpreted according to the biological background (proliferation and cell density)


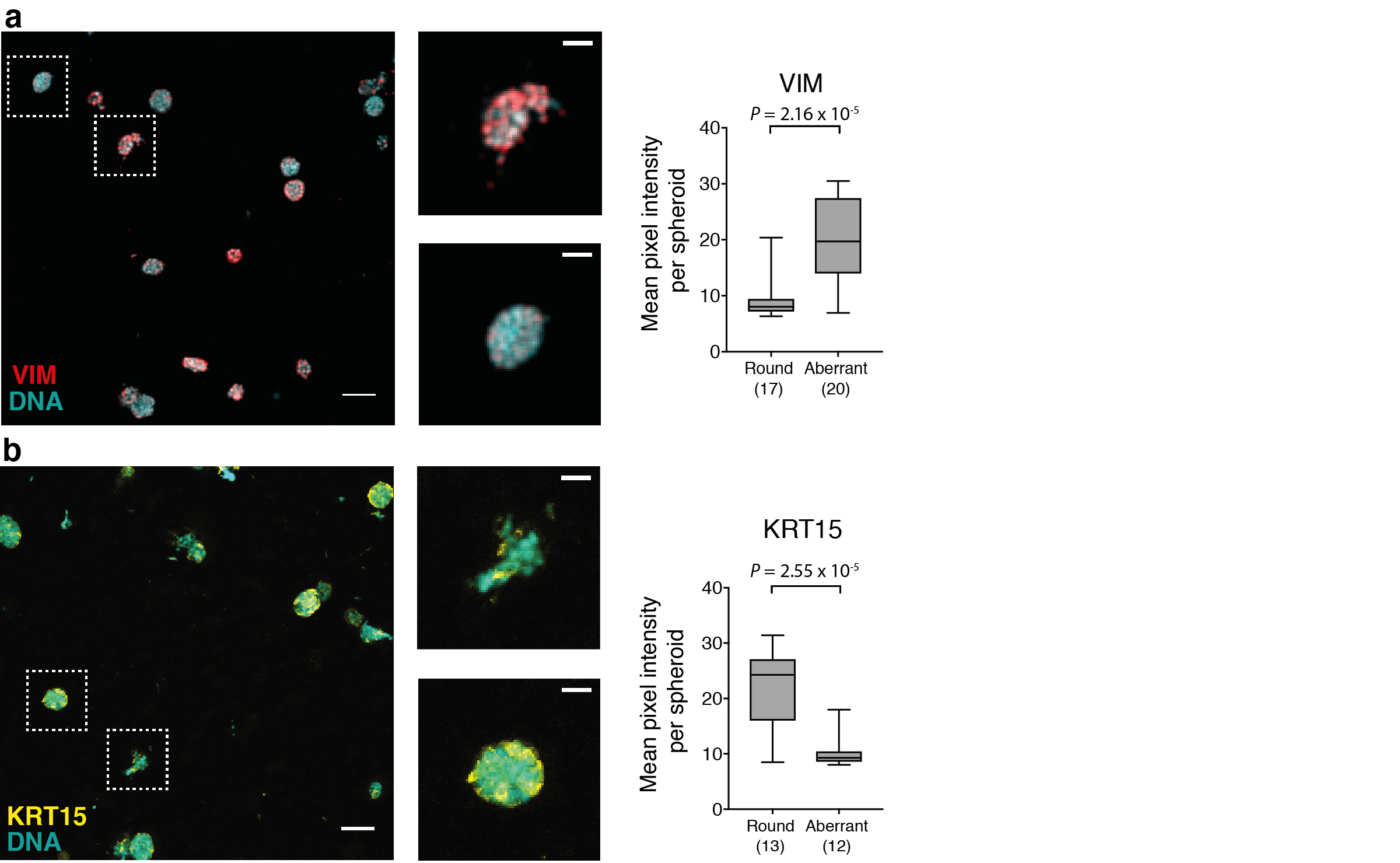


Supplementary Figure 5 | Validation of RNA-seq data by quantitative fluorescence microscopy.

**(a and b)** Immunofluorescence staining with primary antibodies targeting VIM and KRT15 (b). Images represent Z-projections of whole mount spheroid immunofluorescence. Plotted values reflect the mean pixel intensity per classified spheroid of the respective class. Dashed boxes in overview images (scale bar 100 µm) correspond to magnified images beside (scale bar 30 µm). Samples are counterstained with Hoechst dye to visualize nuclei (Hoechst: cyan; KRT15: yellow; VIM: red). (Box plot center-line: median; box limits: first and third quartile; whiskers: min/max values; Indicated *P*-values from unpaired two-tailed Students t-test; Numbers of samples indicated on x-axis under respective class).


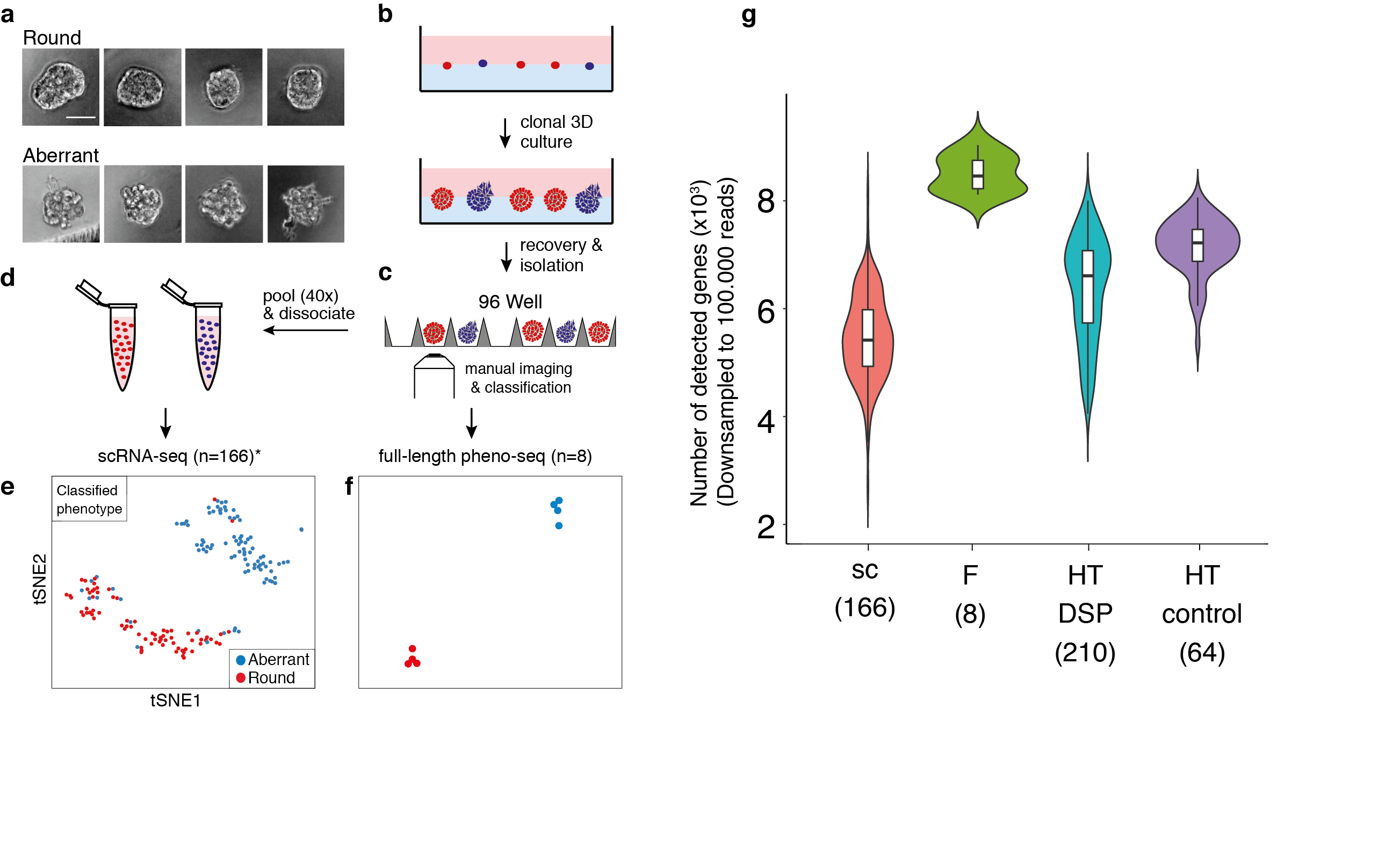


Supplementary Figure 6 | Full-length scRNA-seq and full-length pheno-seq based on manually isolated spheroids.

**(a)** Brightfield images of clonal MCF10CA spheroids (phenotype classes ‘round’ and ‘aberrant’) after isolation from Matrigel (scale bar 50 µm). **(b and c)** Workflow overview for the culture, recovery (b) and isolation (c) of clonal spheroids for the identification of morphology-specific gene expression for full length pheno-seq/scRNA-seq. **(d)** Indirect phenotype – transcriptome correlation by scRNA-seq using cells isolated from multiple (40) spheroids of one annotated morphology phenotype. **(e)** tSNE visualization of 166 scRNA-seq (*cell-cycle corrected) full-length expression profiles of cells derived from manually isolated round and aberrant spheroids. Coloring based on phenotype classification. **(f)** tSNE visualization of 8 full-length pheno-seq expression profiles based on isolated and processed single spheroids. Same coloring as presented in (e). **(g)** Number of detected genes in downsampled scRNA-seq and pheno-seq libraries (sc: scRNA-seq; F: full-length pheno-seq; HT-DSP: high-throughput pheno-seq combined with dithio-bis(succinimidyl) propionate fixation; HT-control: HT-pheno-seq bottom control). Numbers of samples indicated on x-axis under respective strategy.

ô


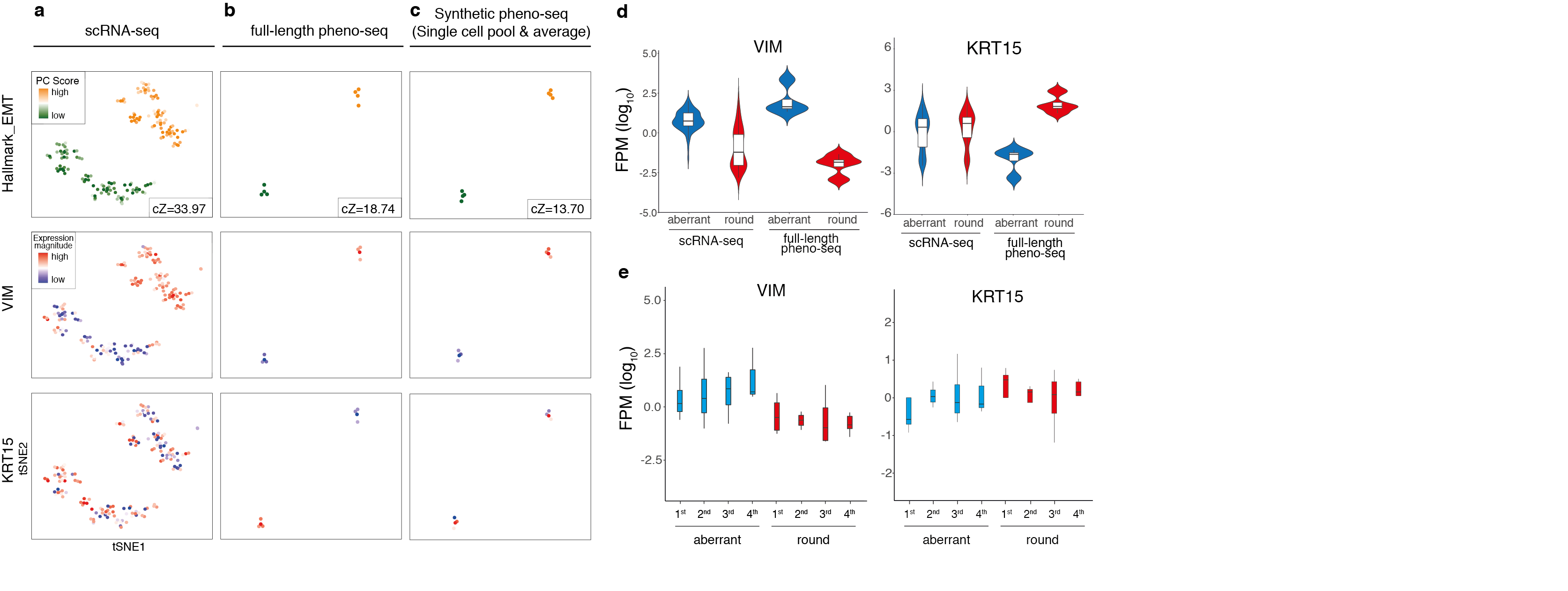


Supplementary Figure 7 | ‘Round’ spheroid marker KRT15 is missed by scRNA-seq

**(a,b,c)**PAGODA tSNE visualizations of MCF10CA scRNA-seq (a), full-length pheno-seq (b) and synthetic pheno-seq (c, based on averaged single cell data). Datasets are colored by PC scores for HALLMARK_EMT gene sets (including associated cZ scores as measure of gene set overdispersion) and by expression magnitude of spheroid phenotype-specific markers VIM and KRT15. **(d)** Violin plots showing expression of individual genes (VIM and KRT15) per identified phenotype-specific clusters for scRNA-seq and full-length pheno-seq. Expression magnitude is plotted as Fragments per Million (FPM, log_10_). Violin-plot center-line: median; box limits: first and third quartile; whiskers: ±1.5 IQR. **(e)** Boxplots showing expression of individual genes (VIM and KRT15) per phenotype-specific clusters for synthetic pheno-seq profiles. Expression magnitude is plotted as Fragments per Million (FPM, log_10_) of four independent randomizations. Boxplot center-line: median; box limits: first and third quartile; whiskers: ±1.5 IQR.


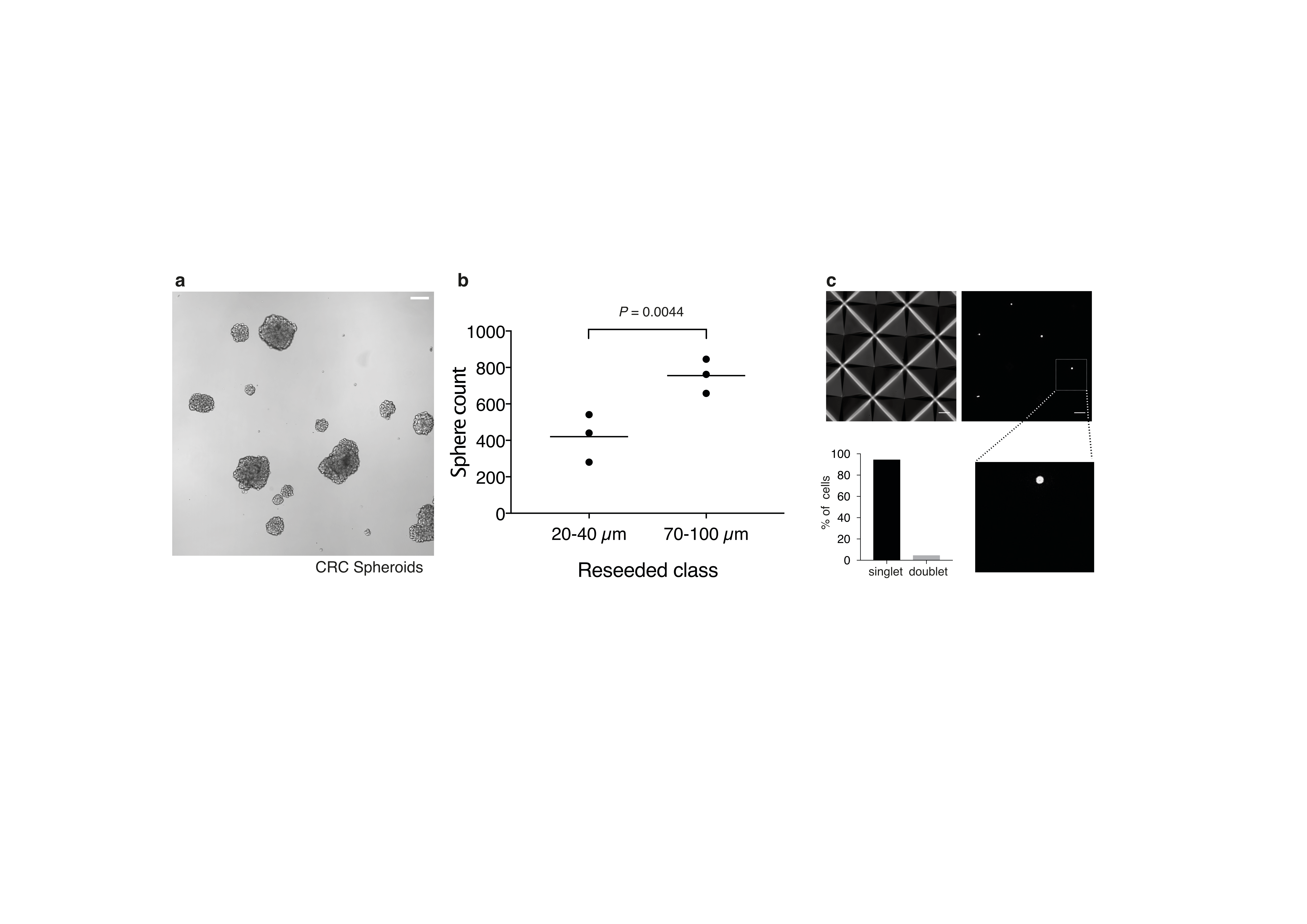


Supplementary Figure 8 | Experimental basis for pheno-seq of a CRC 3D model

(a) Brightfield microscopy image of reseeded and 10 day cultured clonal CRC spheroids derived from a liver metastasis (scale bar 100 µm, example image of class 70-100 µm in (b)). (b) Reseeding assay with cells isolated from distinct spheroid size classes (20-40 µm and >70-100 µm). Plotted are spheroid counts 10 days after reseeding (three replicates, center-line: mean; indicated P-value of paired two-tailed Students t-test). (c) Single-cell seeding efficiency in inverse pyramidal shaped microwells (upper left) assessed by image analysis. Upper right: Example image of CellTracker Red stained cells seeded in microwells (scale bar: 100 µm). Lower right: Magnified image that corresponds to dashed box in upper right image. Lower left: Quantified cell singlets and multiplets after seeding (three wells, four images per well, 70 objects in total).


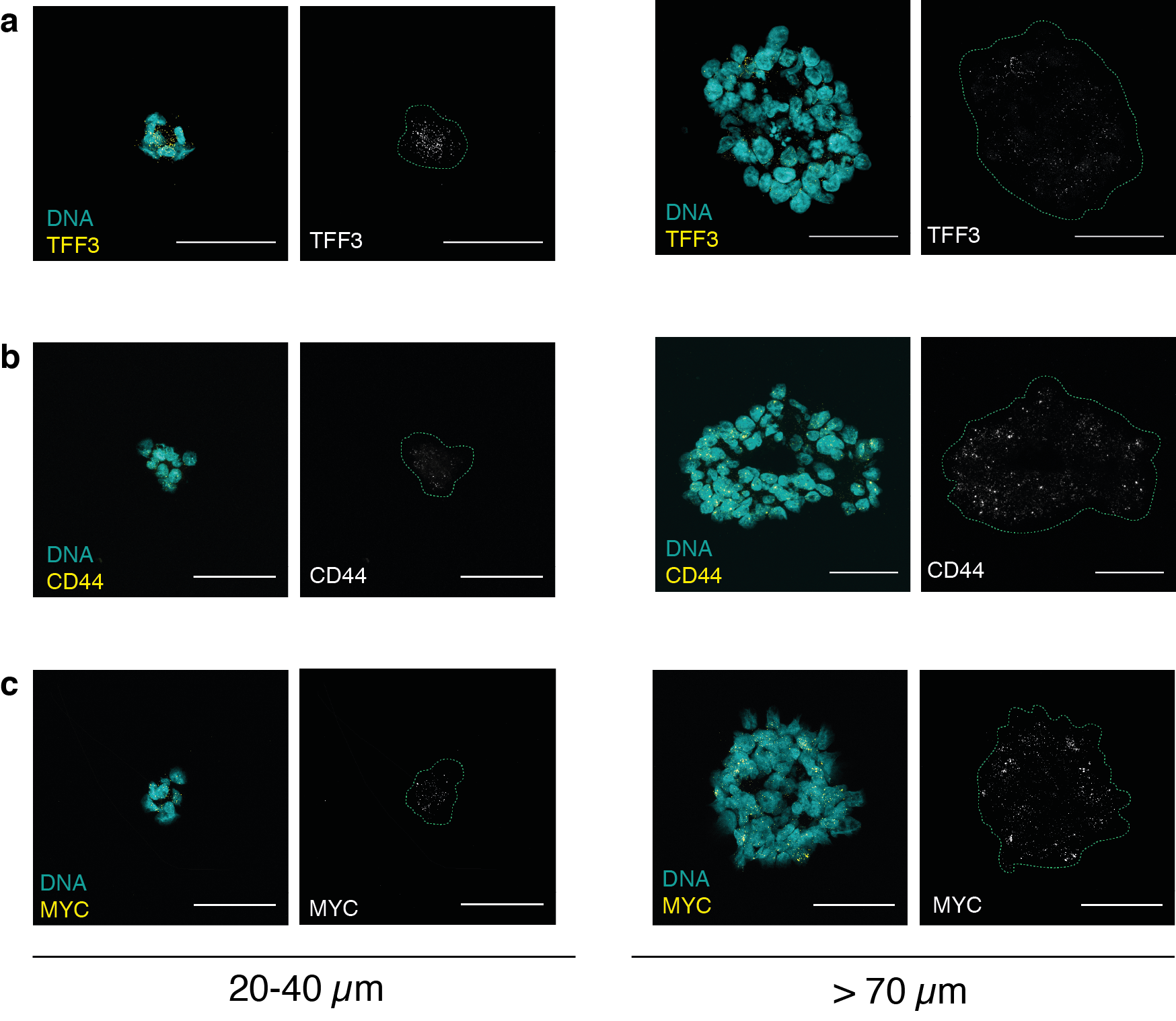


Supplementary Figure 9 | Validation of CRC pheno-seq data by RNA-FISH

(a, b, c) RNA-FISH example images of different spheroid size classes for differentiation marker TFF3 (a) and cancer stem cell markers CD44 (b) and MYC. (c) Z-projections for RNA-FISH staining of big (>70 µm) and small (20-40 µm) spheroids with (left) and without (right) Hoechst counterstain visualization (Hoechst: cyan; RNA: yellow). Dashed line in images without Hoechst visualization represents spheroid border (scale bar 50 µm).

**
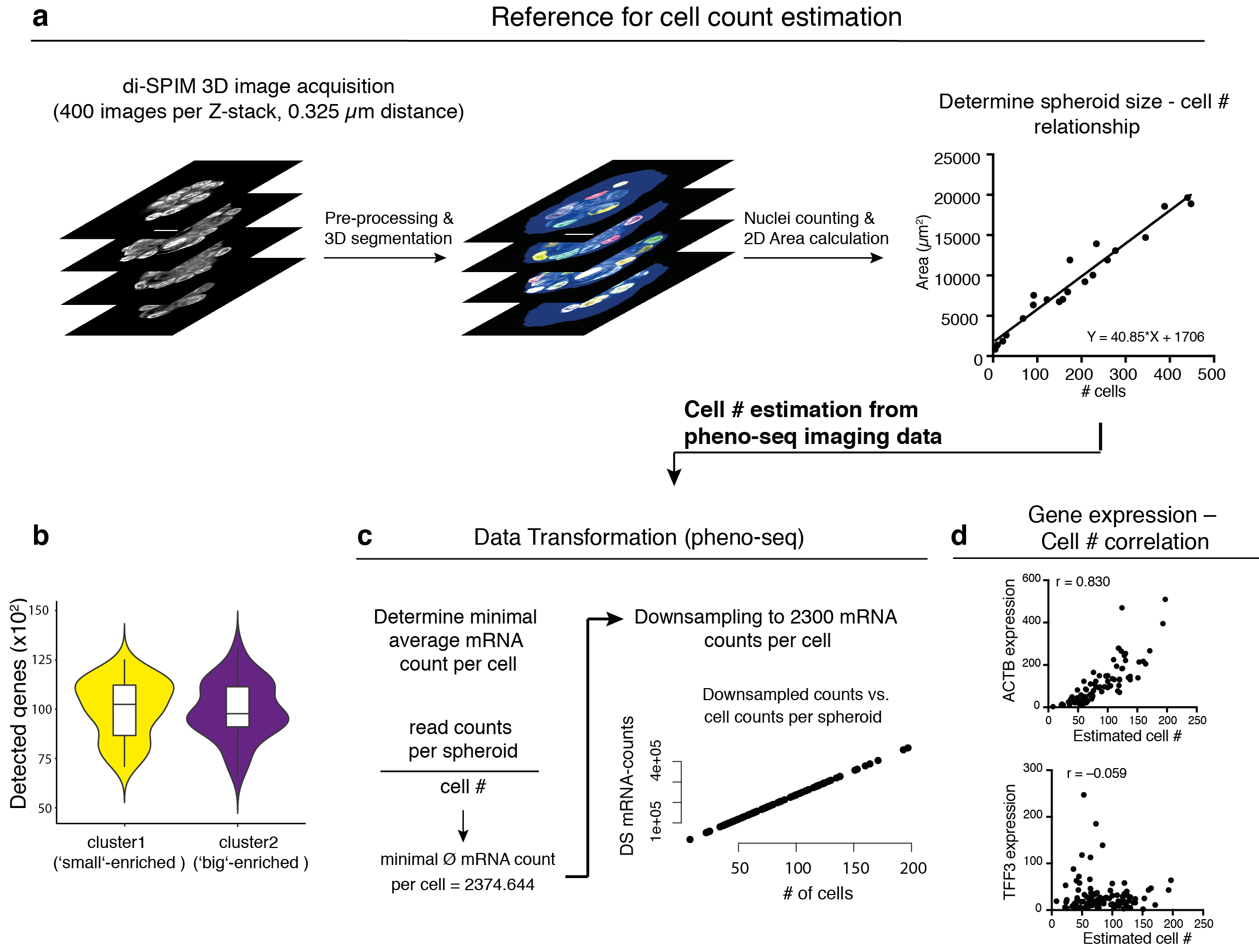
**

Supplementary Figure 10 | Estimation of cell numbers from pheno-seq data using a high-resolution reference dataset for data transformation

**(a)** A two color (Hoechst and CellTracker Red) high-resolution 3D image reference dataset (20 spheroids) is generated by using dual-view inverted selective plane microscopy (di-SPIM). 3D Segmentation and image analysis enables counting of nuclei and the calculated cell number – spheroid size relationship is used to estimate cell numbers from pheno-seq data. **(b)** Violin plots showing detected genes in CRC pheno-seq data per identified cluster. **(c)** Correcting for lost correlation of cell numbers and library complexity by approximating total mRNA abundances in spheroids of different sizes. Raw mRNA counts are divided by estimated cell numbers and the calculated minimal average mRNA count is used to transform the data by downsampling counts to 2300 counts per cell in the whole CRC pheno-seq dataset. Scatter plot showing relationship of estimated cell number and mRNA counts after data transformation. **(d)** Relations of estimated cell numbers and downsampled mRNA counts visualized as scatter plots as well as associated Pearson’s correlation coefficients (r) for housekeeping gene ACTB and differentiation markers TFF3.

**Supplementary table 1 | Dataset overview and sequencing information**

| 3D-culture model | MCF10CA | MCF10CA | MCF10CA | MCF10CA | MCF10CA | CRC spheroid |
| --- | --- | --- | --- | --- | --- | --- |
| Method | scRNA-seq | Synthetic pheno-seq | Full-length  pheno-seq | HT-pheno-seq (control) | HT-pheno-seq (DSP) | HT-pheno-seq |
| Library structure | Full-length  C1 | Full-length  C1 | Full-length  Tube-based | 3`-end  iCELL8 | 3`-end  iCELL8 | 3`-end  iCELL8 |
| Number of samples after library QC | 166 | 8 | 8 | 64 | 210 | 95 |
| Mean total read count per sample | 3,820,057 | 3,685,536 | 9,965,986 | 485,975 | 803,669 | 1,304,480 |
| Mean detected genes (> 0) per sample (all reads) | 8,844 | 15,783 | 12,360 | 8,458 | 8,221 | 9,891 |
| Mean detected genes (> 0)  per sample  (down-sampled to 100k reads) | 5,554 | 13,374​ | 8,411 | 7,051 | 6,377 | 7,412 |

(HT-pheno-seq: high-throughput pheno-seq; control: bottom control with default chip and imaging settings; DSP: Fixation with dithio-bis(succinimidyl propionate crosslinker; C1: Fluidigm C1)

## Supplementary methods

**Assessing single-cell seeding efficiency**

Wells with Hoechst 33258 (1 µg/ml) and CellTracker Red CMPTX (10 µM)-stained single-cells wells were imaged with a 10x/0.30 air objective (Leica HC PL FLUOTAR) of a confocal laser-scanning microscope (Leica SP8) one hour after seeding. MCF10CA (24-well) and colon tumor cell (6-well AggreWell400) images (three independent wells) were analyzed with custom made KNIME image analysis workflows to count seeded cell singlet and doublets.

For quantification of single cell seeding efficiency in both MCF10CA and CRC cultutres, cells were segmented based on the CellTracker signal. First, images were flattened by Gaussian convolution (sigma=1), followed by global thresholding using otsu’s method. Furthermore, a water shedding (default as implemented in Image J) and a minimum filter (min=5 pixels) were applied and cells touching the border were excluded from further analysis. The same workflow was applied to a duplicated set of images, however radially increasing the size of the labels (Max filter node, span 6) after thresholding, resulting in enlarged cell masks. The generated labels were projected on the previously generated original single-cell segmentations.

**Reseeding assay MCF10CA and ‘ilastic’ classification**

Images were acquired on a Zeiss LSM780 Axio Observer confocal microscope equipped with a 10x/0.3 air objective (Zeiss EC PLAN-NEOFLUAR) in brightfield. For quantification of ‘round’ and ‘aberrant’ phenotypes in MCF10CA 3D-cultures we used the random-forest based machine learning software ilastik^49^. A training dataset for the ‘Pixel classification’ option was first generated on randomly seeded and cultured spheroids, whereas classification was based on images derived from independently reseeded cells from round and aberrant 3D phenotypes. Classification was performed in a custom KNIME workflow by applying the trained model to each image.

For quantification of ‘round’ and ‘aberrant’ phenotypes in MCF10CA 3D-cultures, spheroids and background pixel classes were first labeled with the paintbrush tool and classified as ‘round’ and ‘aberrant’. Iterative training allowed stable probability maps that distinguished the three object types (‘round’, ‘aberrant’, ‘background’). For automated analysis of MCF10CA reseeding assays, a custom KNIME workflow loaded a previously trained project file using the ‘ilastik headless node’. Images to be classified were imported into KNIME and classified by applying the trained model to each image. Probability maps for ‘round’ and ‘aberrant’ spheroids were smoothed (manual threshold of 0.5) and a size threshold of 3000 pixels was applied for all segmented objects based on the probability maps. Objects of both classes were automatically counted and assigned to their respective experiment and condition.

**Reseeding assay CRC spheroids**

Images were acquired on a Zeiss LSM780 Axio Observer confocal microscope equipped with a 10x/0.3 air objective (Zeiss EC PLAN-NEOFLUAR) in brightfield. For reseeded colon tumor cells derived from defined size classes ´big´ (70-100 µm) and ´small´ (20-40 µm), 8x8 images per well of the grown spheroids were automatically acquired in 6-well plates (Greiner) using a custom Zeiss VBA macro. All images were analyzed using a custom KNIME workflow to measure spheroid counts per condition.

For quantification of spheroid counts after reseeding of different size classes, spheroid edges were detected using the default function “Find Edges” as implemented in ImageJ. Subsequently, images were manually thresholded (value=20) and neighboring spheroids were separated by applying the default watershedding algorithm to each image as implemented in ImageJ. After segmentation, only segments within the range of 500 to 1x10^5^ pixels (1pix = 0.73 μm) were used and counted for further analysis to exclude single-cells and potential large clusters of spheroids.

**HT-pheno-seq microscopy workflow, image pre-processing and analysis**

For imaging of the iCELL8™ nanowell chip, we used an inverted Leica SP8 confocal microscopy system with an automated stage and the Matrix Screener software extension. In general, also other automated confocal microscopes can be used, but we recommend to use a system where the focus position can be corrected beforehand. A step-by-step microscopy and image analysis protocol can be found in the pheno-seq github repository: <https://github.com/eilslabs/pheno-seq>.

After spheroids have been dispensed into the iCELL8 chip (see Methods), the tightly sealed chip with spheroids centrifuged upside-down to the foil is fixed on a metallic Chip Spinner (TakaraBio) with adhesive tape and placed into a standard microscope plate holder. At this step, it is important to keep the chip upside-down and to keep the orientation of chip consistent for image processing and analysis. Next, a pre-defined 72x72 grid is loaded and the 4 wells at the upper left corner are used for manual focusing and adjusting the position (10x/0.30 air objective Leica HC PL FLUOTAR, 0.9x digital zoom). The ´predictive focus´ option was used to extrapolate the correct focus position for the whole chip. At this step, we recommend to control the focus plane for several positions. In addition, the camera orientation of the used microscope should be also checked beforehand. Excitation was set to 405 and 552 nm and emission filter were set to receive signals between 415 – 485 nm (Hoechst) and 555 – 625 nm (CellTracker Red), respectively. Laser intensity and gain were adapted for every experiment, but the pinhole was set to 5.0 Airy Units permanently. One image contained 512x512 pixels, with 2.53 μm pixel size.

For processing of acquired HT-pheno-seq images, we developed an automated image processing pipeline base don KNIME/ImageJ (see github repository: <https://github.com/eilslabs/pheno-seq>). The two first nodes (“List files”) need to be set to the path that contains the locally saved microscopy data on your computer. The workflow is run until the first node after the second metanode named “crop wells and get correct names” (If the the metanode throws an error, the manual parameter for the global threshold needs to be adjusted). Image names were first transformed so that the order generally matches well locations for barcode assignments as the order of image acquisition deviates from default imaging in the standard system. The resulting output was comparable to the output generated by the standard iCELL8 microscope, although with 2x2 wells per field of view instead of 6x6. In addition, images were rotated by 90^o^ to correct for camera orientation of the Leica SP8 system. Subsequently images were cropped to obtain images containing only one well per image instead of four wells in one field of view. For cropping, a mask was built for each well based on the segmentation of the field of view containing the four most round segments over all positions. Since the microscope can image 4 wells in a manner that only minimal offset between well positioning appeared, this straightforward method allowed cropping of all wells over all positions. In the next step, names of cropped images with single wells were transformed to row/column positions for compatibility with iCELL8-specific barcode assignments. For instance, the four wells of the first top-left image (after re-ordering) were transformed to ‘0_0’, ‘0_1’, ‘1_0’ and ‘1_1’. Further segmentation and analyses were only performed on the cytoplasmic signal (CellTracker Red). The image was first smoothed with a Gaussian convolution algorithm (sigma=5) and then manually thresholded (value=20). Spheroids in close proximity to each other were separated with a watershed algorithm (ImageJ command ‘watershed’, default parameters). Only segments between 25 and 40,000 pixels were considered for further analysis to exclude artefacts and single-cells. Segments touching the border of the image were excluded. Cropped well images were saved automatically in designated locations individually for each channel but also as overlay of the two channels for better visualization in the Shiny app. The images are then converted to .png format using a custom ImageJ macro. In the last step, a .csv file was generated that contained names of all wells containing at least one spheroid. Additionally, calculated 2D features (derived from KNIME image processing node ‘Feature Calculator’) of the corresponding labels were appended (e.g. size and circularity).

The saved .csv file containing spheroid statistics was automatically handled by a custom R script and embedded together with the images in an interactive R/shiny application (PhenoSelect). This allows manual browsing through the acquired images and visualization of spheroids together with their respective image feature statistics. Moreover, the application allowed for visual inspection of the given image features over the whole population, allowing the identification of specific subtypes, e.g. by a particular shape or size. To characterize absolute spheroid sizes, the respective major axis length value (in pixels) was multiplied with the physical length of a pixel in the segmented object. Subtypes of spheroids can be selected by applying different sets of thresholds (e.g. size, circularity) and individual wells can be discarded if necessary (e.g. due to imaging artefacts). The list of selected wells can be saved at any time and also reloaded to proceed with selection at a later time-point. Furthermore, comments can be added to individual wells. Control wells can be selected individually. Once the desired number of wells to be sequenced had been selected, the application generated the ‘filter file’, which is then used to program the iCELL8 dispenser software. In addition, a ´well-list file´ was generated that contained well-barcode assignments for demultiplexing as well as calculated image features for selected spheroids. Finally, we implemented plotting of image features on pre-computed t-SNE maps based on gene expression generated by PAGODA (see below). After sequencing of selected spheroids, this tab enabled integrative analysis for direct association of functional visual phenotypes to transcriptomic heterogeneity.

### Leakage test

Due to the additional centrifugation step to the foil, we assessed potential leakage by dispensing a highly fluorescent solution of PBS + 1 µg/ml fluorescein sodium salt (Sigma) into one half of the nanowells and dispensed only PBS into the other half and into control wells. A dispensing pattern was chosen to generate a maximum number of borders between nanowells filled with fluorescein and those only filled with PBS. Subsequently, the chip was processed and imaged as described above but with laser and filter sets matching the fluorescent properties of fluorescein (λ_ex_ 460 nm; λ_em_ 515 nm).

The acquired images were processed for well assignment, segmentation and cropping by the HT-pheno-seq pre-processing workflow and average fluorescence intensity was measured for every well independently. Average fluorescence intensity values ranging from 0 to 255 (8 bit) were color coded and plotted onto a 72x72 grid resembling the iCELL8 chip layout using a custom R script.

**Cell count determination by light sheet imaging and 3D segmentation**

To estimate the cell number in spheroids in HT-pheno-seq experiments based on acquired images, we generated a high-resolution 3D reference dataset to determine the linear relationship of size (area) and cell count. CRC spheroids were stained with 1 µg/ml Hoechst and CellTracker Red CMPTX (10 µM) for 3 hours and isolated and fixed as described above. Subsequently, spheroids were mounted in 2% low-melting agarose (Sigma) and 3D images were acquired using a Dual-View Inverted Selective Plane Illumination Microscope (ASI di-SPIM) using Nikon 40x/0.80W NA NIR-Apo water dipping objectives. Dual view raw data was processed to generate isotropic images at a resolution of 0.325px/µm (400 images per Z-stack, 0.325 µm distance). Pre-processing and 3D segmentation were performed with a custom KNIME workflow. To count nuclei from the high-resolution light sheet microscopy dataset, a 2D projection of the smoothed CellTracker image produced a mask that was generated individually for each spheroid. The obtained 2D mask covering the spheroid was applied to all individual slices, in order to count nuclei only within this area to exclude artifacts. Single-cells were first segmented and counted individually for each slice. Subsequently, overlapping cells were separated by a watershed algorithm (KNIME node: Waehlby Cell Clump Splitter). Furthermore, the 2D segmentations were used as ‘seeds’ for three-dimensional Voronoi segmentation, in order to obtain a three-dimensional segmentation that accounts for cells spanning multiple slices. Cell counts (without correction for potential changes in ploidy) and corresponding 2D spheroid dimensions were exported from KNIME and further analyzed and plotted using a custom R script. Briefly, we first converted the respective pixel numbers for minor and major axis of all 2D spheroid masks to metric distances. Facing the challenge of variable spheroid morphologies, we further approximated the spheroid size as the product of its biggest and smallest diameter (i.e. the minor and major axis). Subsequently, we plotted the approximated size of every spheroid against its respective cell count determined by 3D segmentation in KNIME. A linear model was fitted through the points and the obtained slope was used to calculate cell number estimations for HT-pheno-seq experiments.
